# Animal products, diseases and drugs: a plea for better integration between agricultural sciences, human nutrition and human pharmacology

**DOI:** 10.1186/1476-511X-10-16

**Published:** 2011-01-20

**Authors:** Olav A Christophersen, Anna Haug

**Affiliations:** 1 Ragnhild Schibbyes vei 26, 0968 Oslo, Norway; 2 Department of Animal and Aquacultural Sciences, Norwegian University of Life Sciences, 1432 Ås, Norway

## Abstract

Eicosanoids are major players in the pathogenesis of several common diseases, with either overproduction or imbalance (*e.g. *between thromboxanes and prostacyclins) often leading to worsening of disease symptoms. Both the total rate of eicosanoid production and the balance between eicosanoids with opposite effects are strongly dependent on dietary factors, such as the daily intakes of various eicosanoid precursor fatty acids, and also on the intakes of several antioxidant nutrients including selenium and sulphur amino acids. Even though the underlying biochemical mechanisms have been thoroughly studied for more than 30 years, neither the agricultural sector nor medical practitioners have shown much interest in making practical use of the abundant high-quality research data now available. In this article, we discuss some specific examples of the interactions between diet and drugs in the pathogenesis and therapy of various common diseases. We also discuss, using common pain conditions and cancer as specific examples, how a better integration between agricultural science, nutrition and pharmacology could lead to improved treatment for important diseases (with improved overall therapeutic effect at the same time as negative side effects and therapy costs can be strongly reduced). It is shown how an unnaturally high *omega*-6/*omega*-3 fatty acid concentration ratio in meat, offal and eggs (because the *omega*-6/*omega*-3 ratio of the animal diet is unnaturally high) directly leads to exacerbation of pain conditions, cardiovascular disease and probably most cancers. It should be technologically easy and fairly inexpensive to produce poultry and pork meat with much more long-chain *omega*-3 fatty acids and less arachidonic acid than now, at the same time as they could also have a similar selenium concentration as is common in marine fish. The health economic benefits of such products for society as a whole must be expected vastly to outweigh the direct costs for the farming sector.

## Background

The world is faced with unprecedented challenges in the environmental sector, especially as a consequence of large anthropogenic emissions of greenhouse gases (including methane and N_2_O from agriculture), but also because of unchecked deforestation and soil erosion affecting large geographical areas. On top of this, we have serious pollution problems that may not only threaten non-human species (such as the polar bear), but also can represent a serious genetic hazard to *Homo sapiens *himself, since many of the pollutants concerned are either highly mutagenic or weaken important antimutagenic defense mechanisms, and they interact with several other chemical mutagens (*e.g. *from tobacco, alcohol and mutagenic drugs) associated with our modern lifestyles.

The most serious immediate threat to us might conceivably come from various positive feedback regulatory mechanisms inherent in the global climatic system, leading to the possibility of a sudden, major change in global temperature and/or in the geographical distribution of arid and humid zones. It is known that such sudden changes of the global climate, as exemplified by the end of the Younger Dryas [1], have occurred repeatedly during the fairly recent geological past [2], most recently about 4.2 thousand years ago when the Akkadian Empire [3] and the Egyptian Old Kingdom [4] both collapsed. During the last event, the change of global temperature was much more modest than had been the case at the end of the Younger Dryas. But there was a major change in rainfall over vast areas in the Middle East and North Africa [3-5], and the Sahara region was permanently changed into a desert [6].

The world is also faced with soaring food prices [7] and very serious challenges in the medical sector, not only in the poor countries [8], but also as a consequence of those changes that are taking place in the age structure of the populations of many industrial countries, at the same time as a number of not acutely lethal non-infectious diseases, such as diabetes type 2 and asthma, have become much more prevalent than they were before. The health sector and the environmental sector are, however, competing with each other for the same limited economic and manpower resources, and it is difficult to see how this situation will change in the foreseeable future. The question may thus be raised, whether it will be economically and politically possible, even for the most affluent countries in North America and Europe, to solve both challenges simultaneously. And who should be left back on the sinking ship, the Alzheimer patients or the global environment, if there is not enough room for both in the lifeboats?

This means that it may be necessary also for the health sector, including the worldwide community of medical scientists, to take its part of the burden, if it shall be possible to avert worldwide economic, social and political collapse as a result of global climatic catastrophe and attendant famine on a scale (as measured by the total number of victims) that might turn out to be unprecedented in the history of our species. There may not be enough resources available to avert total environmental and genetic disaster unless the health sector worldwide can be made vastly more cost-efficient than it is today. But doing so is not something that can be achieved by medical scientists and other health professionals acting alone (*e.g. *by finding new and better drugs), since better prophylaxis of several important diseases will not be possible unless the nutritional quality of the human diet can be made much better than is often the case today. This applies not only to poor countries, but to many of the rich ones as well. It is therefore a great challenge to agricultural scientists, food scientists and nutrition scientists to develop methods and strategies by which agriculture indirectly may make it easier for the world to confront the problems in the environmental sector - by providing foods with better composition and thus reducing the costs needed for health care.

This challenge relates equally to the animal food and to the plant food sectors, with their associated industries as well as to agriculture itself. It will be up to the politicians and international and state regulatory agencies to implement the necessary changes, in particular by imposing new regulatory standards for the composition of commonly consumed foods (like poultry meat, pork meat and eggs). In this article, we will not discuss the issues of local and global resource constraints, skewed global distribution of limited resources, nor the adverse ecological side effects, like deforestation and emission of greenhouse gases, that attend food production in both rich and poor countries. We will limit our attention to the composition of some of the quantitatively most important animal foods, *viz. *poultry meat, pork and eggs. It will be shown how improving the composition of these foods may lead to better prophylaxis of several important diseases. Hopefully this can also make a significant contribution to improved treatment of some of the diseases concerned (with an improved ratio between therapeutic effects and negative side effects), while perhaps also making therapy economically more cost-effective than now. Examples, based on our own research, show how it is practically feasible (and not too expensive) to obtain such changes in the composition of agricultural products that for medical reasons are needed. But there are also other methods that may be used to obtain similar results. However, we consider it to lie beyond the scope of the current discusson to survey all of the methods that in theory might be used and to compare them as regards their practical feasibility, or from the point of view of agricultural economics.

### Animal products and health: selenium-rich and long-chain *omega*-3-fatty acid-rich fish versus arachidonic acid-rich meat

The market demand for foods with a nutrient composition adjusted to optimize human health and life expectancy is growing. Such foods are often referred to as functional foods. Many foods have so many beneficial health effects even without any modification during production or processing that they might well be referred to as natural functional foods. The composition of certain other foods has been so much modified as a result of industrial methods of food production or processing that they well might deserve to be referred to as 'antifunctional foods', since their negative health effects may outweigh the positive ones. Some foods can not be characterised either as uniquely functional or antifunctional because their positive health effects may be dominating only for certain groups of consumers (depending on their specific dietary requirements, the nature of their most important disease problems and the composition of the rest of their diet); the negative health effects of the same type of food may be the dominating ones for other consumer groups.

Sea fish products can be regarded as natural functional foods; their protective effects can be explained partly by high natural concentrations of long-chain *omega*-3 fatty acids (especially in fatty fish), taurine [9-12] and selenium. Meat is usually regarded as less beneficial than fish for protection against cardiovascular diseases and cancer. Chicken meat, nevertheless, is commonly regarded by both health professionals and the general public as a healthful type of meat; it is well-liked, and the consumption is increasing [13]. Chicken meat is lean, protein-rich and a good source of important micronutrients, such as zinc and vitamin B_12_, and conditionally essential nutrients, such as nucleotides (mainly in the form of RNA and DNA) and carnitine. Chicken meals are often preferred to a fish dinner, even in such cases when the consumer knows that fish is more healthful regarding both *omega*-3 fatty acids and selenium. However, the concentration of selenium (Se) in chicken breast meat in Scandinavia is only about 0.01 mg/100 g [14,15], while fish fillet contains about 3 to 4 times as much [14,16].

In chicken thigh meat, the total amount of the very long-chain *omega*-3 fatty acids eicosapentaenoic acid (EPA), docosapentaenoic acid (DPA) and docosahexaenoic acid (DHA), as given by the Danish food composition table, is only about 0.06 g/100 g, while in cod it is 0.26 g/100 g and in fatty fish such as salmon it is about 2.8 g/100 g [14]. The concentration of arachidonic acid (AA) in chicken is 0.09 g/100 g, in cod 0.02 g/100 g and in salmon 0.09 g/100 g [16]. Thus the ratio between AA and the sum of EPA+DPA+DHA is 10-40 times higher in chicken than in fish. These figures show that nowadays fish fillet is not only a better source of Se, but also a much better source for *omega*-3 fatty acids than chicken meat, and that fish gives a much lower relative load of AA.

The fatty acid composition and Se concentration in chicken meat depend largely on the composition of the diet fed to the birds. Feed composition affects the fatty acid composition of the product [17], and it has earlier been shown that feeding poultry with *omega*-3 fatty acids from rapeseed oil and linseed oil improved the ratio between *omega*-6 and *omega*-3 fatty acids and increased the concentrations of *alpha*-linolenic acid (ALA), EPA, DPA and DHA in broiler thigh muscle [18]. Furthermore it is known that dietary supplements of Se-enriched yeast increase the Se concentration of the chicken meat [19] and other animal products [20]. Commercial chicken feed is cereal-based (wheat, barley or corn), and the added fat is mostly rendered fat and vegetable oils, giving a diet with a high ratio of *omega*-6 to *omega*-3 fatty acids. The diet eaten by poultry in their natural habitats consists of seeds, plants, insects etc., providing plenty of minerals, micronutrients and plant antioxidants and a much higher proportion of *omega*-3 fatty acids compared to *omega*-6 fatty acids than in those feed mixtures that are now commonly used in commercial poultry production.

### What is the role of endogenous synthesis of long-chain polyunsaturated fatty acids in humans, compared with intake from the diet?

The human organism can, like other mammals, use fatty acyl elongases and desaturases to convert the 18C PUFAs linoleic acid (LA), ALA and *gamma*-linolenic acid (GLA) into long-chain PUFAs. But attempts to measure the rate of endogenous synthesis of AA, EPA and DHA in humans, compared to the magnitude of ordinary dietary intakes of long-chain PUFAs (in populations with a mixed diet), have given discrepant results with most studies [21-23] showing poor and some extremely poor [24] conversion of 18C PUFAs into long-chain PUFAs: < 5-10% for EPA and 2-5% for DHA [22].

It is difficult to understand, however, how women who are lactovegetarians or vegans can give birth to babies with normal brains if the endogenous capacity of DHA synthesis from ALA in humans is not larger than some of the experimental studies (especially from North America) have shown. There are several hundred million poor people worldwide (probably more than 1 billion altogether) who for purely economic reasons can not afford to eat more than minuscule quantities of animal foods. It may be legitimate to ask the question, how it is possible for the children of all these people to grow up without serious learning problems and intellectual deficits due to inadequate DHA supply for the growing brain, unless the human capacity to make DHA from ALA is better (perhaps locally in the brain, if not necessarily at a systemic level outside the central nervous system) than some of the best studies until now do indicate. It is evident that the health of several children must be damaged owing to several other forms of malnutrition that have a large global prevalence and also affect brain development, like zinc [25,26], iodine [27,28], iron [29,30], folate [31] and vitamin B_12 _[32] deficiencies. But we might expect the situation worldwide to be even worse if the effect of widespread DHA deficiency on brain development comes in addition to all the other deficiencies that we know exist.

In a traditional Mediterranean diet with high intake of olive oil, much of the total intakes both of LA and ALA would be expected to come from olive oil, which has an LA/ALA concentration ratio that for genetic reasons is variable, but often may be around 10/1 [33,34]. A study of the fatty acid composition of human blood samples from the population in Crete showed, however, that plasma lipoprotein cholesteryl ester contained 31.0 +/- 2.7% oleic acid, 41.9 +/- 3.7% LA and only 0.9 +/- 0.5% ALA [35]. Unless there is a highly preferential incorporation of LA instead of ALA into plasma lipoprotein cholesteryl esters (which can not be a priori excluded), these figures suggest faster metabolic degradation of ALA than of LA in humans, most likely by about a factor of 4. This could be either because of faster *beta*-oxidation or peroxisomal oxidation of ALA, compared to LA, or because of faster conversion of ALA into long-chain PUFAs.

The latter hypothesis would help to solve the paradox of how it may be possible for the children of vegan and lactovegetarian women to grow up without serious cognitive deficits resulting from inadequate DHA supply to their growing brain. If correct, it also suggests that humans may have high capacity to convert ALA into long-chain *omega*-3 PUFAs, even in situations where the intake of long-chain PUFAs from animal foods is fairly high. If this explanation for the observations from Crete is correct, it implies that at least one of the elongases or desaturases must have high substrate specificity, with the rate of *omega*-3 fatty acid conversion being much higher than for its *omega*-6 fatty acid analogue. This would not appear unreasonable from the point of view of evolutionary ecology, given the large size of the human brain and the very high normal concentration of DHA in the membrane lipids of vertebrate brains.

The explanation for the highly divergent observations concerning the possible capacity of humans to convert 18C PUFAs into long-chain ones is not well understood. Some causes of variations in the capacity for converting ALA into DHA are known either from studies in animals or humans (or both): there is a gender difference with adult female rats [36,37] and adult female humans [36] both having better capacity to convert ALA into DHA compared to adult males. And it has been found in rats that vitamin A deficiency is associated with impairment of this conversion in animals fed an ALA-poor diet [38], while the opposite effect of vitamin A deficiency was observed in the liver when the intake of ALA was high [39]. However, neither of these factors can explain why ALA conversion into DHA might be poorer in Canada [24] than in Crete.

It is not unreasonable that part of the explanation for individual or geographic differences in the capacity to convert 18C PUFAs into long-chain ones could be negative end-product regulation of the expression and/or activity of some of the fatty acyl elongases or desaturases, in particular at the first two steps of the pathway of metabolic conversion of 18C PUFAs into long-chain ones. If this is correct, a high dietary intake of long-chain PUFAs from animal foods would be expected to lead to a corresponding depression of the capacity to convert 18C PUFAs from plant foods into long-chain PUFAs. This mechanism has been observed in rat liver, though not in the rat brain [40]. Yet end-product inhibition of the expression or activity of these enzymes can not explain the observations from Crete, when the LA/ALA ratio of human blood plasma cholesteryl esters is compared with that of olive oil, since most people there do not subsist on vegan or lactovegetarian diets, and the average intake of long-chain PUFAs from animal foods is most likely fairly high.

#### Could geographical differences in micronutrient intake lead to geographical differences in the extent of ALA conversion into EPA and DHA, with this conversion being better in warm countries than in Canada and northern Europe?

We have suggested as an alternative (but still entirely hypothetical) explanation that there might be large geographical variations, due to differences in soil chemistry, in the intake of some micronutrient (presumably some essential trace element) that is needed for the metabolic conversion of 18C PUFAs into long-chain ones, with the average intake of the micronutrient concerned being much lower in Canada than in Crete [41]. The trace element vanadium (V) could be a possible candidate, since it can occur in several oxidation numbers [42], and its bioavailability for uptake into plant roots, similarly as for chromium (Cr) [43], depends heavily on redox equilibria in the soil [44]. Vanadate (VO_4_^---^) is somewhat more soluble and more bioavailable for the plants (because of the chemical similarity between vanadate and phosphate, as regards their binding to membrane transporters), compared to vanadyl ions (VO^++^). Vanadyl ions are only slightly soluble [45], are tightly bound to soil organic matter [46,47], and have most likely no root uptake system of their own (for active transport into plant roots).

Vanadium concentrations in plant foods, *e.g*. cereal grains, are typically even lower than the Cr concentrations in areas with much organic matter in the topsoil, such as the wheat-growing areas of central North America [48], this in spite of V having a larger average concentration (60 ppm) than Cr (35 ppm) in the rocks of the upper continental crust [49]. More than half of 34 samples of wheat grain from 12 different locations in North America were found to contain less than 6.5 ppb (microgram/kg) V, and the highest level was 20.0 ppb [48]. In the same study, Cr concentrations ranged from 3 to 43 ppb with a mean of 17 ppb [48]. But unrefined plant foods that have been produced in warmer countries where the soil contains little organic matter (because of faster microbial degradation when the soil temperature is higher), such as unrefined cane sugar, can have much higher concentrations both of Cr and V, compared to North American wheat [48]. In a study of Cr concentrations in molasses and unrefined, brown, and highly refined sugar from several countries, the mean values obtained were 266 +/- 58 ppb for the molasses, 162 +/- 36 ppb for the unrefined sugar, 64 +/- 5 for the brown sugar, and 20 +/-3 ppb for the refined (white) sugar [48]. Similarly, barley from Iraq has been reported to contain about 100-200 times more Cr [50] than barley from Finland [51]. Barbados brown sugar was reported to contain 400 ppb V, compared to 2 ppb V in white sugar [48]. Fish meal appears to be an exceptionally good source of vanadium, with 2700 ppb having been reported for herring fish meal [48]. This may reflect the relatively high dissolved V concentration in seawater (much higher than for Cr) [52], with vanadate probably being taken up by algae by the same ATP-dependent membrane transport system (or systems) that is used for uptake of phosphate, arsenate and perhaps selenite.

Vanadium can thus be expected to be more bioavailable for uptake by the plants on Crete than in Canada. The climate on Crete is warmer and the soil therefore contains less organic matter than in Canada; at the same time the average soil pH is also most likely fairly high. And V has general chemical properties that presumably could make it suitable to function as a catalyst for some of the kinetically more difficult redox reactions taking place in living organisms, like fatty acyl desaturation.

If this vanadium hypothesis (for explaining poorer conversion of ALA into DHA in Canada compared with Crete) can be confirmed, an important implication would be that pregnant and lactating women in Canada would probably have a much higher requirement for long-chain PUFAs from their diet compared to, say, women in Nigeria or Tanzania. But it also means for the latter women that if they as a consequence of poverty can not obtain enough long-chain PUFAs from animal foods, it may be even more important than for Canadian women that their intake of ALA is adequate. Also the LA/ALA ratio in their total diet should not be too high, to ensure enough endogenous synthesis of DHA, compared with the growth requirement of their foetus or baby (for ensuring normal development of the brain of the latter). It could possibly also mean that there might be a need for internationally accepted regulatory guidelines, making it mandatory for all companies selling vegetable fats and oils directly to consumers on markets in low-income countries to ensure that the *omega*-3 fatty acid (ALA or sum EPA + DPA + DHA) concentrations of their products should not be below some lower threshold value and that the LA/*omega*-3 fatty acid ratio similarly should not exceed some upper threshold value, with both threshold values being determined by the regulatory agency (or agencies) concerned. Taking unrefined palm oil as an example, this could in principle very easily be achieved by mixing the palm oil with a modest quantity of linseed oil and/or good quality fish oil.

### Effects of membrane lipid fatty acid composition on membrane fluidity and on the rates of electron transport through chloroplast and mitochondrial membranes

In their natural habitats, herbivorous or omnivorous animals acquire much of their intake of ALA from green leaves, which normally have a surplus of ALA over LA in their membrane lipids [53]. This is a great paradox, given the enormous oxidative stress normally associated with photosynthesis (*i.a. *as a consequence of the very high O_2 _partial pressure, higher than in ambient air, and of the very abundant formation of singlet oxygen [53]). But it can probably be explained as a result of the effect of different fatty acids on the fluidity properties of the thylakoid (inner chloroplast) membrane lipids. These have a high concentration of ALA, making the membrane more fluid, especially at low temperature, compared with what would result from a similar concentration of LA, which is important for the cold tolerance of the plants [54-56]. Higher membrane fluidity permits faster diffusion of plastoquinone between different protein complexes in the thylakoid membranes, which means faster electron transport through the photosynthetic apparatus. Plastoquinone has a function in the chloroplasts similar to that of ubiquinone in our mitochondria [53]. Fast electron transport through the thylakoid membranes seems in this particular context to be an even more important priority for the plants, compared to antioxidant protection (which they can achieve by several other methods).

The freezing point of vegetable fats and oils decreases - for a given average number of C atoms per fatty acyl group - as the average number of double bonds per fatty acyl group is enhanced. Marine oils with high concentrations of the long-chain *omega*-3 PUFAs EPA and DHA (*e.g. *cod-liver oil) have especially low freezing temperatures. The effect of different PUFAs on the fluidity properties of biological membranes is probably very similar to what can be observed in more easily observable edible fats and oils. The large average number of double bonds per fatty acyl group that is typical of marine animals (with much long-chain *omega*-3 PUFAs) can therefore be interpreted, at least in part, as an adaptation to low ambient temperature, making it possible for fishes like capelin (*Mallotus villosus*), salmon, herring and cod to swim fast (with rapid electron transport through their mitochondrial respiratory chains), even when the seawater temperature is low.

#### Why is there so much DHA in the human brain, testicles and spermatozoa?

It is not unreasonable to suggest that the same mechanism also can explain why there is so much DHA in the membrane lipids in mammalian brains [57,58], as well as in the testicles [59] and spermatozoa [60,61].

For the brain, there is probably a double advantage to be gained, if the Ohmian resistance to lateral electron transport through the inner mitochondrial membrane can be minimized by improvement of the fluidity properties of the membrane. One the one hand, this must be expected to help to enhance the maximal mitochondrial ATP production capacity per gram tissue when some part of the brain is activated. This, in turn, may presumably help to enhance the rate of information processing in the brain, when some special part of it is activated. On the other hand, it must also be expected to help to decrease the rate of mitochondrial production of reactive oxygen species (ROS) for a given rate of ATP production. A reduction of the rate of mitochondrial ROS production when the fluidity of the inner mitochondrial membrane is improved is most likely achieved by a double mechanism:

(a) by counteracting accumulation of electrons at the top of the respiratory chain (in complex I) because they can flow with less Ohmian resistance from complex I to cytochrome *c *oxidase when the membrane is more fluid; this means reduction of the rate of superoxide anion radical generation by reaction between molecular O_2 _and redox-labile groups (iron-sulphur and/or flavine) in complex I (that happens when the latter are in a reduced state),

(b) by helping the cell to maintain a given rate of ATP production at a lower intramitochondrial O_2 _partial pressure (because of faster electron supply to cytochrome *c *oxidase). This will also help to reduce the rate of superoxide anion radical production by reaction between O_2 _molecules and complex I.

In the testicles, germ cells multiply at a very high rate before they mature into fully differentiated spermatozoa. Cell growth is highly ATP-dependent; at the same time there must also be good reason to protect the DNA of the germ cells as well as possible from damage caused by ROS. Improving the fluidity of the mitochondrial inner membranes of the germ cells might then presumably be a good strategy for minimizing the ratio between the rates of mitochondrial ROS production and mitochondrial ATP production.

After the spermatozoa have been discharged in the female genital tract, there will be a fierce competition - in a true evolutionary, Darwinian sense - to be the first one to reach their target, which is a competition that only one of them (among the huge number of sperms) can win. In species where multiple matings involving more than one partner are common, it must be expected that males that produce spermatozoa that for genetic reasons have larger ATP production capacity and therefore can swim faster will have a marked Darwinian fitness advantage compared to such males who produce spermatozoa with mitochondria that have a lower ATP production capacity. The same mechanism would, moreover, also be expected to favour males who have better-functioning sperm mitochondria because they are younger and therefore have less age-related mutations in their sperm mitochondrial DNA.

Enhancement of the rate of ROS-induced mutagenesis in these organs is a serious matter, even more so when it happens in the testicles than when it happens in the brain. When the rate of mitochondrial mutagenesis is enhanced in the brain, a likely consequence will be higher risk and earlier onset of various age-related degenerative brain diseases, such as Alzheimer's disease and Parkinson's disease. However, when the same happens in the testicles, it can not be expected that nuclear DNA will be spared when the rate of ROS production is enhanced in the mitochondria owing to abnormal composition of the mitochondrial inner membrane lipids. The consequence will then be enhancement of the rate of ROS-induced germline DNA mutations. This will directly affect the health of countless future generations, especially when it occurs at a population level (because of a change in the composition of the average diet eaten by the whole population) and not affects just a few unfortunate individuals (as in the case of occupational exposure to ionizing radiation for astronauts or workers in uranium mines). In a worst case scenario, it may even be the survival of our species that could be at stake if the total burden of germline mutagenesis becomes too high in both men and women.

#### Possible role of mitochondrial membrane fluidity in cardiac and skeletomuscular diseases, in neurodegenerative diseases including peripheral neuropathies, and in diabetes type 2

Most of the other cells in the human body lack the unique capacity for DHA accumulation in their membrane lipids that we find in the central nervous system, the retina and the testicles. But it must be expected that even in these more normal cell types (considering the fatty acid composition of their mitochondrial membrane lipids), there must be an effect of the dietary *omega*-6/*omega*-3 fatty acid ratio, probably for both the 18C and long-chain PUFAs, on the *omega*-6/*omega*-3 fatty acid ratio and the fluidity properties of their inner mitochondrial membrane. This would also influence the Ohmian resistance to electron transport from complex I to cytochrome *c *oxidase. Enhancement of this resistance would directly lead to increased "damming up" of electrons in complex I, which must in turn be expected (other factors being equal) to lead to enhancement of the rate of intramitochondrial ROS production.

The fluidity of mitochondrial membranes depends also on other factors than their fatty acid composition, and some of the changes associated with normal aging may apparently lead to enhanced membrane rigidity, or reduced fluidity [62]. In the heart, it has been found that age-associated mitochondrial membrane changes include increases in membrane rigidity, cholesterol, phosphatidylcholine, *omega*-6 PUFA and 4-hydroxy-2-nonenal, and decreases in *omega*-3 PUFA and cardiolipin [62]. It might be speculated that the age-associated enhancement in the *omega*-6/*omega*-3 PUFA ratio, even when the fatty acid composition of the diet is constant, is something that happens as a result of preferential degradation of *omega*-3 PUFAs when mitochondrial ROS production is enhanced as a direct consequence of the aging process [63]. We have ourselves similarly found in experiments with broilers that the *omega*-6/*omega*-3 long-chain PUFA ratio of the meat depends upon the selenium (Se) intake of the animal, with better Se status being associated with enhancement of the DHA concentration in the meat. This is most likely a consequence of improved protection of DHA against degradation by processes of lipid peroxidation [18]. A similar protective effect of good Se status against DHA degradation by peroxidation may in principle be expected to occur also in aging humans, while enhancement of the rate of mitochondrial ROS production would be expected to have an effect in the opposite direction in both species.

The above-mentioned effects of aging on the properties of membrane lipids in the heart have been shown in animal studies to be exaggerated by a diet rich in AA [62]. They have profound consequences for the efficacy of membrane proteins involved in ion homeostasis, signal transduction, redox reactions and oxidative phosphorylation [62]. However, some of the age-related detrimental changes may be beneficially modified by dietary intervention [62]. Diets rich in *omega*-3 PUFA have been reported to reverse the age-associated membrane *omega*-6/*omega*-3 PUFA imbalance and also the age-associated dysfunctional Ca^++ ^metabolism, at the same time as they improve the efficiency of mitochondrial energy production and also the tolerance of ischemia and reperfusion [62,64].

Another group has also observed in animal experiments that a lower *omega*-6/*omega*-3 fatty acid ratio in the myocard was associated with faster recovery of mitochondrial energy metabolism and myocardial pump function during reperfusion following experimental ischemia [65]. The tolerance of the myocard to ischemia followed by reperfusion is thus improved by a reduction in the dietary *omega*-6/*omega*-3 PUFA ratio, at least as far as the long-chain PUFAs are concerned (*i.e. *the AA/(EPA + DPA + DHA) ratio) [65,66]. But one may in principle expect that a reduction of the LA/ALA ratio of the diet also could have an effect going in the same direction - with ALA having a similar effect also in our mitochondria, when it replaces LA, as it has in the thylakoid membranes of the plants.

Similar effects may in principle be expected also in other organs, where enhanced mitochondrial ROS production as a consequence of abnormally rapid (or pathological) mitochondrial DNA aging could represent an important part of the pathogenetic mechanism of perhaps several different degenerative diseases, most likely including type 2 diabetes [67]. It is not implausible that this also could play a role in the etiopathogenesis of skeletomuscular diseases often affecting elderly people, including pains associated with skeletal muscle spasms or overload, and perhaps also the degenerative changes affecting cartilage in patients suffering from osteoarthritis. In both cases, it is not unreasonable that changes in inner mitochondrial membrane lipid composition could interact synergistically with mitochondrial DNA mutations (and sometimes also cytokines) as causes of enhanced mitochondrial ROS production.

Furthermore, enhancement of mitochondrial ROS production must be expected to interact synergistically with factors such as Se, glutathione, taurine, carnosine or other antioxidant nutrient depletions that lead to impairment of the antioxidant defense capacity of the muscle or cartilage cells. This would apply also for cells in the brain (which might be important in neurodegenerative diseases such as Alzheimer's disease) and in peripheral nerve fibres (which might be important in diabetic peripheral neuropathy and other cases of C-fibre dysfunction), as well as for the *beta*-cells in the pancreas, which means it might well also be important in the etiopathogenesis of type 2 diabetes. There is good reason to hope that multifactorial therapeutic interventions for reducing the pathologically elevated mitochondrial ROS production while optimizing the cellular capacity for scavenging ROS might be helpful in all of the above-mentioned diseases, at least for secondary prophylaxis by reducing their rate of further progression, but in some cases (*e.g. *in common pain conditions associated with C-fibre dysfunction, and perhaps type 2 diabetes) also by partial symptom reversal.

While oleic acid replacement of LA would be expected to have the opposite effect on membrane fluidity to that happening when ALA or some long-chain PUFA replaces LA in the same membrane lipid position, it might be speculated that such detrimental effects of oleic acid on membrane fluidity could be partly or entirely compensated for by oleic acid substitution not only for PUFAs, but also for saturated fatty acids in the membrane lipids. Oleic acid substitution for a saturated fatty acid, like stearic acid (with the same number of C atoms), in a position not normally occupied by PUFAs would presumably lead to reduction of the membrane fluidity. The same can also be expected to happen when a saturated fatty acid with shorter chain length replaces one with longer chain length, as in the case of palmitic acid replacing stearic acid. It seems, however, that there is little research literature dealing with these questions. It is an important topic probably deserving far more intensive research attention than it has received so far.

### Dietary AA/(EPA + DPA + DHA) and ALA/LA ratios, gene expression and eicosanoid biosynthesis

While 18C fatty acids are found in both animal and plant foods, with a large proportion of the total dietary intake coming from edible fats and oils, long-chain PUFAs come nearly exclusively from animal foods (especially meat, fish and eggs) and dietary supplements made from seafish, such as cod-liver oil and fish oil capsules. The ratio between dietary intakes of long chain *omega*-6- and long chain *omega*-3 fatty acids is therefore in large measure determined by the long chain *omega*-6/*omega*-3 fatty acid concentration ratio of the different animal products that we commonly eat, as well as by the frequency of eating animal foods with either high or low long-chain *omega*-6/*omega*-3 fatty acid ratios.

For people living on such mixed diets that are common in the industrial countries, endogenous synthesis of long-chain PUFAs from 18C PUFAs is also important, and the ratio between the concentrations of long chain *omega*-6- and long chain *omega*-3 fatty acids found in our tissue lipids will be similarly dependent on both the magnitude of dietary intakes of long-chain PUFAs as such and the magnitude of dietary intakes of LA and ALA. For people especially in poor countries who for economic reasons can not afford to eat much animal food, it must be expected that endogenous synthesis of long-chain PUFAs from LA and ALA will normally dominate over dietary intakes of long-chain PUFAs as such. It must therefore be very important for the general health situation in these countries that the edible fats and oils eaten by less affluent people should have an optimal fatty acid composition in which the *omega*-6/*omega*-3 ratio is not too high. As earlier explained, this applies also to the normal development of the brain of foetuses and children.

The ratio between long-chain *omega*-6 and long-chain *omega*-3 fatty acids is now considered to be so high in many of the western societies that it substantially increases the mortality and/or morbidity associated with several non-communicable diseases. This includes some of the leading causes of death in the countries concerned, such as coronary heart disease and malignant arrhythmia [68,69]. This happens not only because of enhanced incidence for some of the diseases concerned, *e.g. *for acute thrombotic events, but also because of symptom aggravation or more rapid progression of already established disease (*e.g. *intensification of skeletomuscular pains or more rapid progression of colon cancer as a consequence of prostaglandin overproduction).

One of the explanations for the harmful effect of over-abundance of *omega*-6 fatty acids in the diet has already been mentioned, *viz. *the effect of changes in the AA/(EPA + DHA) ratio in the lipids of the inner mitochondrial membrane on the fluidity properties of the membrane. A high AA/(EPA + DHA) ratio leads to stiffening of the membrane and enhanced Ohmian resistance to the transport of electrons from complex I to cytochrome *c *oxidase. This will in turn lead to enhancement of the rate of mitochondrial ROS production. Another important reason is the different effects that *omega*-6 fatty acids and *omega*-3 fatty acids have on gene expression. *Omega*-3 fatty acids hinder the expression of inflammatory genes [70], whereas *omega*-6 fatty acids have proinflammatory effects [70].

Inflammation can take place within the vascular walls and plays a role in modulating the effect of insulin and control of inflammatory gene expression and lipid metabolism [70]; this is important not only in connection with diabetes type 2, but also as a part of the disease mechanism during progression of atheromatosis/atherosclerosis [70]. *Omega*-3 fatty acids decrease the endothelial responsiveness to proinflammatory and proatherogenic stimuli by modulating the expression of adhesion molecules and cytokines important for the processes collectively denoted as "endothelial activation" [71]. Studies on postprandial inflammation (which is now considered an independent risk factor for diseases such as atherosclerosis and insulin resistance) indicate that each meal triggers an inflammatory response, and that the ratio between *omega*-6 and *omega*-3 fatty acids is an important determinant of the magnitude of this postprandial inflammatory response [72].

It should also be noted that another fatty acid, *viz. *oleic acid, has been reported to have protective effects similar to those which the long-chain *omega*-3 fatty acids have been demonstrated to have on endothelial cells, namely by reducing rates of intracellular generation of reactive oxygen species (ROS) [73] and counteracting the activation of nuclear factor-*kappa*B [74]. This may probably help to explain the strongly protective effect against myocardial infarction that was found for a modified Mediterranean diet in the Lyon trial, compared to such "prudent diets" that were then commonly recommended to patients suffering from coronary heart disease [75]. In this case, olive oil had been partly replaced by a margarine rich in rapeseed oil, which was rich in oleic acid as well as ALA.

#### Effect of the dietary AA/(EPA + DPA **+ **DHA) and LA/ALA ratios on the balance between thromboxane and prostacyclin biosynthesis and on the total rate and effects of prostaglandin biosynthesis in diseases other than cardiovascular disease

Another important reason why overconsumption of AA is harmful (especially when combined with low dietary intakes of EPA and DHA) is the tendency for prostaglandin and thromboxane A_2 _overproduction in disease situations, when the absolute intakes of arachidonic acid (AA) and/or LA, the dietary ratio of AA to the sum of EPA and DHA, or the ratio of LA to ALA in the diet are too high. In a recent study of the specificities of enzymes and prostanoid receptors toward EPA-derived, 3-series versus AA-derived, 2-series prostanoid substrates and products, the largest difference was seen with PG endoperoxide H synthase-1, also called COX-1 [76]. Under optimal conditions, it was found that purified COX-1 oxygenates EPA at a rate which is only 10% of the rate for AA, while EPA significantly inhibits AA oxygenation by COX-1 [76]. 2-fold to 3-fold higher activities or potencies with 2-series versus 3-series compounds were observed with COX-2, PGD synthase, microsomal PGE synthase-1, and EP1, EP2, EP3 and FP prostanoid receptors [76]. Surprisingly, it was observed that AA oxygenation by COX-2 is only modestly inhibited by EPA; COX-2 exhibits a marked preference for AA when EPA and AA are tested together [76]. Also unexpectedly (and contrary to the earlier belief that thromboxane A_3 _(TxA_3_) is inactive), it was found that TxA_3 _is about equipotent to TxA_2 _at the TP*alpha *receptor [76]. These observations predict that increasing the EPA/AA ratios in the phospholipids of human cells would dampen prostanoid signalling, the largest effects being on COX-1 pathways involving PGD, PGE, and PGF. Production of 2-series prostanoids from AA by COX-2 would be expected to decrease in proportion to the compensatory decrease in the AA content of phospholipids that would result from increased incorporation of *omega*-3 fatty acids such as EPA and DHA [76].

It should be noted that even in the COX-2 pathway, one must expect much less stimulation of the EP1, EP2 and EP3 receptors if one starts with EPA rather than AA. This is due to a multiplicative effect of less rapid conversion of EPA into PGH_3_, less rapid conversion of PGH_3 _into PGE_3 _and less potency of PGE_3 _at the receptors (EP1, EP2 and EP3), compared with AA, PGH_2 _and PGE_2_. However, as far as COX-2 is concerned, it is important to recognize that AA competes not only with EPA and DHA, but also with LA, ALA and oleic acid for incorporation in the same positions in membrane lipids. Enhancement of the EPA and DHA concentrations at these positions will therefore not be attended by a proportional reduction of the AA concentration. Thus the best strategy for avoiding prostanoid overproduction in disease situations where COX-2 is important must be to reduce the intake of AA, rather than just enhancing the intakes of EPA and DHA.

It should, furthermore, be taken into consideration that not only do AA, EPA and DHA compete with each other for binding to COX-1 and COX-2 (with EPA and DHA inhibiting the conversion of AA into PGH_2 _and AA and DHA inhibiting the conversion of EPA into PGH_3_). but also 18C unsaturated fatty acids (especially the 18C PUFAs, but also oleic acid) can bind to COX-1 and COX-2, albeit considerably weaker than the 20C and 22C PUFAs, and can thus function as competitive inhibitors of the conversion of 20C PUFAs into prostaglandins and thromboxanes [77-80]. Even though the 18C unsaturated fatty acids are fairly weak inhibitors of 20 C PUFA oxidation by cyclooxygenases, it should not be forgotten that they are are much more abundant than the latter, especially LA and oleic acid. A high total intake of 18C PUFAs and oleic acid may thus help to antagonize some of the harmful effects of over-intake of AA from animal foods with unbalanced *omega*-6/*omega*-3 fatty acid ratios. This is not just because of their competitive displacement of long-chain PUFAs from corresponding positions in the membrane lipids, but also because of their effects as COX inhibitors. This may be considered one of the (perhaps few) beneficial effects of diets rich in LA in some, but not all disease conditions. One should, on the other hand, not forget the important role played by dietary LA as a precursor used for endogenous synthesis of AA.

The long-chain PUFAs of animal foods appear to be associated mainly with membrane lipids (plus blood plasma lipoproteins), while the concentration of long-chain PUFAs in the triglycerides of adipose tissue such as lard is surprisingly low [16]. The somewhat paradoxical conclusion may be reached that it would probably be better for many patients, when overproduction of prostaglandins is a major problem (*e.g. *in various pain conditions), and when the *omega*-6/*omega*-3 ratio of some animal food (*e.g. *pork meat) is too high, to eat meat products containing much adipose tissue rather than lean meat. It is thus possible that efforts to breed animals (*e.g. *swine) with proportionately less adipose tissue compared with muscle (because this was thought to be better for the health of the consumer) may have been largely futile, as far as the intended health effects are concerned. It would probably be much better, if we want to minimize prostaglandin production (*e.g. *in a patient with chronic pain, or a patient with metastatic colon cancer), to recommend animal foods that have a low *omega*-6/*omega*-3 fatty acid ratio at the same time as the proportion of adipose tissue to muscle is high. This could be done especially when the adipose tissue from the animal has a low concentration of LA with higher relative concentrations of oleic acid, ALA and saturated fatty acids (*e.g. *meat from sheep or goats that have been slaughtered during the autumn after they have been fattened on mountain pastures during the whole summer), while animal adipose tissue containing too much LA relative to the sum of ALA, oleic and stearic acid (since stearic acid can partly be converted into oleic acid following intestinal absorption in the human body) should not be similarly recommended.

LA has been reported to function as a much stronger inhibitor of COX-2 than COX-1 [78,79], while for ALA there is much less difference between its COX-2 and COX-1 inhibitory activities [78]. Since endothelial COX-2 is important for prostacyclin (PGI) synthesis [81], while platelets contain only COX-1, one must expect that a high total intake of LA or a high dietary LA/ALA ratio will depress the synthesis of prostacyclin in the endothelium much more than it depresses the synthesis of thromboxanes in the platelets. This must in turn be expected to enhance the risk of adverse thrombotic events, *e.g. *in the brain, given the strong prothrombotic effect of thromboxanes [82] and the strong antithrombotic effect of the prostacyclins [82]. Here, the effect of a high total dietary intake of LA is similar to that of a high dietary AA/(EPA + DHA) ratio, and also similar to the effect of some selective COX-2 inhibitors, such as celecoxib and valdecoxib, that have now been retracted from the market because of their cardiovascular side effects [83].

### Should enhanced intake of oleic acid be recommended as a strategy for antagonizing the effects of too much arachidonic acid in western diets?

In theory, it should be possible to use enhanced dietary intakes of 18C polyunsaturated fatty acids (LA and ALA) or oleic acid to displace AA, rather than reducing the AA intake, for reducing its concentration in the membrane lipids of human cells. At the same time the 18C unsaturated fatty acids will also function as competitive inhibitors of the cyclooxygenases. It is conceivable that a high intake of LA for this reason could be protective against cardiovascular disease in populations who consume too much AA and too little EPA and DHA, even though the role of LA as a precursor for endogenous synthesis of AA would rather appear to suggest the opposite. This applies also to the stronger inhibitory effect of LA on COX-2 compared with COX-1, as explained above, which is another possible reason why a high intake of LA might enhance the risk of adverse thrombotic events rather than decrease it. Another important disadvantage of enhancing the total intake of other polyunsaturated fatty acids (even LA with only two double bonds in its side chain) for reducing the AA concentration of human membrane lipids, rather than reducing the dietary intake of AA, is that this method must be expected to lead to enhanced lipid peroxidation, other factors being equal.

This objection, however, does not apply to enhancing the dietary intake of oleic acid as a possible strategy for reducing the AA concentration of the membrane lipids in leukocytes, platelets, endothelium and other human cells. This is because oleic acid with only one double bond is not easily peroxidised, like all polyunsaturated fatty acids (with the vulnerability to oxidative attack increasing with the number of double bonds per molecule). Instead it is very resistant to non-enzymatic oxidative attack. Enhancement of the oleic acid/polyunsaturated fatty acid concentration ratio (*e.g. *the oleic acid/LA ratio) at a given position in phosphatidylcholine or other lipid molecules will therefore make the lipid molecule concerned less vulnerable to non-enzymatic oxidation. This applies both to lipid molecules in membrane structures inside the cells (*e.g. *in the mitochondria) and to those in plasma lipoproteins. There is also good reason to expect that it may apply not only to the susceptibility of lipid molecules to peroxidation by free radical reactions, but also to their susceptibility to oxidation by peroxynitrite, which is a potent and highly reactive oxidant, but not itself a free radical (although it may give rise to free radicals by secondary reactions).

#### Proatherogenic and mutagenic effects of products of lipid peroxidation

In the blood and the vascular walls, it appears now to be well documented that oxidized LDL is much more atherogenic than non-modified LDL [84,85]. But it must also be expected that enhanced lipid peroxidation everywhere (both in the blood, in the vascular walls and in any other cell type) will lead to enhanced production of aldehydes that are mutagenic, such as malondialdehyde [86], crotonaldehyde [87], acrolein [88], and 4-hydroxynonenal [89]. This must in turn be expected to lead to enhancement of the rate of accumulation of deleterious mutations in both nuclear and mitochondrial DNA. Increase in the rate of mutations in the nucleus enhances the risk of cancer; increase of the rate of mutations in mitochondrial DNA means abnormal enhancement of the rate of mitochondrial DNA aging [63,90].

It is possible, however, that the mutagenic effects of some of the aldehydes concerned could be much larger in the mitochondria than in the nucleus because of less efficient DNA repair in the mitochondria, which have a more restricted repertoire of DNA repair mechanisms compared to the nucleus. These mutations therefore could be more important as causes of accelerated aging rather than as causes of cancer. 4-Hydroxynonenal has, moreover, also been reported to function as a potent inducer of the immunosuppressive [91] and fibrogenic [92] cytokine TGF-*beta*, which might be highly relevant in the pathogenesis of arteriosclerosis.

#### Role of selenoprotein P as a protectant against plasma lipoprotein peroxidation, atheromatosis and thrombosis

As far as the blood lipids are concerned, the rate of LDL oxidation presumably depends on several other factors in addition to its fatty acid composition (*i.e. *its PUFA/oleic acid ratio, with LA being most important among the PUFAs for people living on a normal European or North American diet). The rate of production of peroxynitrite when superoxide anion radical from endothelial NAD(P)H oxidase reacts with NO (also produced by endothelial cells, which is something we shall return to and discuss in more detail later) is probably one of the most important factors governing the rate of LDL oxidation, if not the most important one. Anything that can enhance the activity of the endothelial NAD(P)H oxidase must also be expected to enhance the rate of LDL oxidation, while anything that can be done in order to correct pathologically elevated endothelial ROS and peroxynitrite production will be helpful for protection against atheromatosis, arteriosclerosis and thrombosis.

Another factor that is most likely very important for the rate of LDL oxidation, is the activity of the antioxidant protective enzyme selenoprotein P [93,94]. This protein has a function in extracellular environments similar to that which GPx-4 (also called phospholipid hydroperoxide glutathione peroxidase) has inside the cells, reducing organic hydroperoxides associated with membrane molecules or with plasma lipoproteins [93,94]. But GPx-4 and selenoprotein P use different reductants, since GPx-4 uses GSH, while selenoprotein P prefers reduced thioredoxin [95].

It should be noted that human extracellular fluids also contain thioredoxin reductase [96,97], needed for regeneration of reduced thioredoxin after its oxidation in the selenoprotein P reaction. Thioredoxin reductase is both a flavoprotein and a selenoprotein [98,99]. It contains a selenocysteyl group and a cysteyl group in adjacent positions [99], making it possible for these groups to form chelate complexes with heavy metal atoms. Thioredoxin reductase is therefore extremely vulnerable to inhibition by toxic metals. It is more sensitive by a factor of 1000 or more than GPx-1 and glutathione reductase to inhibition by aurothioglucose or another organic gold complex called auranofin [99]. While GPx-1 is a selenoprotein containing isolated selenol groups (without opportunities for chelate formation), glutathione reductase is an ordinary dithiol protein, with which chelates can be formed by simultaneous coordination of a heavy metal atom with 2 sulphur atoms.

The human selenoprotein P gene codes for 10 selenocysteyl groups per molecule [100]. But it is often not fully saturated with Se, since inadequate Se intake can lead to premature termination of translation for some of the molecules [101-103]. It is apparently not known if there is only one active site for hydroperoxide reduction per molecule, one for every Se atom (unless the site concerned is blocked by a heavy metal atom), or an intermediate number of active sites (more than 1, but less than 10).

Selenoprotein P is a glycoprotein [102] that contains also many basic aminoacyl groups (lysyl and histidyl groups) [104] and many cysteyl groups [105]. It is bound to the surface of endothelial cells [106,107] and to LDL [108], most likely as a result of electrostatic attraction between positively charged aminoacyl groups on the selenoprotein P molecule and negatively charged groups, *e.g. *phosphate groups in phospholipids, both in LDL and on the surface of the endothelial cells, as well as in heparin [104,109]. The binding of selenoprotein P to LDL can presumably explain why the blood becomes Se-depleted when LDL is removed from the plasma of hypercholesterolemic patients by LDL apheresis [110,111]. Selenocysteyl and cysteyl groups are found in such relative positions that the selenol and thiol groups can be oxidized and form at least two selenenylsulfide linkages per molecule [112]. It would be expected that the selenocysteyl and cysteyl groups concerned also may bind toxic heavy metals avidly, forming very stable chelate complexes with the heavy metal atoms. This is confirmed by observations, showing strong binding of toxic heavy metals, such as silver, mercury and cadmium, to selenoprotein P [113]. Selenoprotein P was found to bind the metals concerned, even when they had already formed complexes with selenide ions before binding to the protein [113].

The concentration of selenoprotein P in human blood plasma depends on the Se intake [114,115], with a Se intake of at least 100 microg/day being needed for saturation in adult persons [116]. The Se intake in most parts of Europe is less than that needed for saturation of blood plasma with selenoprotein P [117]. But the concentration of selenoprotein P in blood plasma can also be changed because of disease, such as prostate cancer [118] or sepsis [119].

It must be theoretically expected that there will be a synergistic (multiplicative) interaction between low Se intake (leading to undersaturation of the blood plasma with selenoprotein P), large exposure to toxic heavy metals (which may be expected to lead to simultaneous inhibition of selenoprotein P and extracellular thioredoxin reductase), a high dietary LA/oleic acid intake ratio and high rates of superoxide anion radical production from endothelial NADPH oxidase as causes of more rapid LDL oxidation - with a high rate of LDL oxidation leading in turn to high rates of atheromatosis development. Against this background it is not unreasonable to speculate that excessive exposure to a number of toxic metals from a wide range of different sources may have been one of the main causes of the post-war epidemic of coronary heart disease both in North America and Western Europe. These include lead from car exhaust and from drinking water (especially in the British Isles), as well as mercury and silver from dental amalgam fillings and cadmium from acid rain, commercial fertilizers and tobacco smoke.

All the above-mentioned toxic metals would be expected to bind strongly to the chelate-forming selenol and thiol groups in both thioredoxin reductase and selenoprotein P (and also, albeit not equally strongly, to chelate-forming thiol groups in thioredoxin). It is possible that their relative importance as causes of enhanced LDL oxidation and atheromatosis, both at the individual level and that of entire populations, may depend less strongly on differences in their relative binding strength to these enzymes than on differences in their abundance. If this hypothesis is correct, it means that lead, which is the most abundant of these toxic metals when considering both its average abundance in the Earth's continental crust [49] and as an environmental pollutant, could have been more important than any other toxic metal as a contributory cause of LDL oxidation, atheromatosis and coronary heart disease. This hypothesis would appear to be in reasonably good agreement with what is known about the historical curves both for coronary heart disease mortality and for the use of lead as an additive in gasoline in Western Europe, compared to North America. The use of lead as an additive in gasoline started earlier and ended earlier in the United States than it did in the countries of Western Europe. And the epidemic of coronary heart disease has followed a similar time course with both its start and its culmination occurring earlier in the United States than in Western Europe.

### Redox regulation of prostaglandin biosynthesis

The rate of prostaglandin (PG) biosynthesis is regulated at two consecutive enzyme reaction steps, first at the level of liberation of eicosanoid precursor fatty acids by hydrolysis of membrane lipids (which is often catalyzed by phospholipase A_2_), and next at the level of the cyclooxygenase (COX, or prostaglandin H synthase) reaction, where precursor fatty acids (such as AA or EPA) are converted into the corresponding PG endoperoxide. For example, prostaglandin H_1 _(PGH_1_) is formed from dihomo-*gamma*-linolenic acid, while PGH_2 _is formed from AA and PGH_3 _from EPA. PGHs are themselves unstable, but are rapidly converted by other enzymes to form other prostaglandins (such as PGE, PGD, PGF_*alpha *_or PGI) or thromboxanes.

There are different isozymes of phospholipase A_2 _with different localization (intracellular or extracellular) and regulation, some being activated by Ca^++^, while others are Ca^++^-independent. Some of these enzymes are activated, or their expression is enhanced, by oxidative stress [120,121], which for a number of different reasons will commonly accompany inflammatory reactions [90,122]. This is one of the reasons why the synthesis of prostaglandins or other eicosanoids (such as leukotrienes) is enhanced in all inflammatory diseases, including allergic diseases and asthma (where leukotrienes are very important).

The cyclooxygenases (COX-1 and COX-2) must be oxidized for activation, and H_2_O_2_, organic hydroperoxides and peroxynitrite can all function as activators [77,90,123-125]. These activators are scavenged by the group of selenoproteins called glutathione peroxidases (GPx) [126,127], which are potent inhibitors of cyclooxygenase activation [77]. But activation of COX molecules will simultaneously start a slower process of suicidal inactivation of the same enzyme molecules [77], which means that each COX molecule can on average only make a limited number of PGH molecules. When GPx counteracts activation of this enzyme, it will simultaneously also inhibit its suicidal inactivation, similarly as has been observed with various biological and synthetic antioxidants [77] that not only inhibit COX activation (because they may reduce the formation of hydroperoxide activator molecules or scavenge peroxynitrite), but also inhibit the irreversible suicidal inactivation of COX.

The main reason why it is still possible for prostaglandin production to be much enhanced during inflammatory reactions, in spite of the limited number of PGH molecules that can be made per COX molecule before the latter is inactivated, is the possibility of upregulating expression of COX-2 (*i.e. *enhancing the production of new enzyme molecules) under such conditions when the rate of its suicidal inactivation is enhanced. The expression of COX-2 in leukocytes is under multiple regulation by several different transcription factors, including the oxidatively regulated [128] transcription factor nuclear factor-*kappa*B (NF-*kappa*B) [129,130], and also other oxidatively regulated transcription factors. So when oxidative stress enhances the rate of irreversible suicidal inactivation of COX-2, it will simultaneously enhance the rate of production of new enzyme molecules.

Low Se intake is associated with reduced activity of GPx in many cell types and organs [126]. This may in turn lead to eicosanoid overproduction because of the combination of more phospholipase A_2 _expression or activation, more rapid COX activation and enhanced expression of COX-2. The same must also be expected to happen when cells are undersaturated with reduced glutathione (GSH), that functions as the reducing substrate for GPx [126]. But since GPx displays *tert-uni *ping pong kinetics [126], and 2 GSH molecules are consumed for each molecule of oxidizing substrate (*e.g. *H_2_O_2_) consumed in the same reaction, the rate of oxidizing substrate removal (at a given concentration of the latter) depends on the second power of the GSH concentration, while it depends only on the first power of the concentration of the enzyme. It must therefore be expected that overconsumption of AA, low intake of Se and GSH depletion will interact synergistically with each other as causes of prostaglandin overproduction, especially during inflammatory conditions where COX-2 expression is enhanced.

It may also be theoretically expected that GSH depletion can be even more important than poor Se status as a cause of prostaglandin or thromboxane overproduction. The latter can potentially lead to thrombotic events, such as brain stroke, and GSH depletion can easily develop in disease situations, especially because of the combination of reduced food intake and enhanced protein catabolism. It can not be expected that this situation will be improved by giving the patients large doses of drugs, such as acetaminophen (paracetamol), that are partly metabolized by forming conjugates with glutathione or other sulphur amino acid derivatives [82].

#### Can interactions between nutritional factors and alcohol and a biphasic effect of alcohol itself on the blood plasma GSH concentration help to explain why moderate alcohol consumption protects against cardiovascular mortality in some countries while excessive alcohol consumption enhances it in Russia?

Alcohol abuse, especially when combined with a poor diet and/or disease leading to enhanced protein and sulphur amino acid catabolism [131-135], can not be expected to make the situation any better for patients suffering from prostaglandin overproduction because of GSH or other antioxidant nutrient depletion (which might be very common, especially in such cases where there is a synergistic interaction between alcohol abuse and poverty as causes of poor health). Alcohol abuse can deplete the liver of glutathione by a combination of different mechanisms [136,137]. These include acute inhibition of glutathione synthesis [136-138] and enhanced GSH efflux to the blood [136-138], but most likely also enhanced excretion of GSSG through the bile, similarly as happens after exposure of the liver to other oxidant stressors [139,140], when the alcohol-induced oxidative stress in the liver [141,142] becomes too high because of enhanced ROS production from several different sources [143-146].

It is possible, however, that moderate consumption of alcohol, especially when taken in combination with protein-rich meals and when the antioxidant nutrient status is good, may have a positive effect on blood GSH concentrations and hence on the GSH status in cells in other parts of the body, outside the liver. This could happen not only because of the stimulating effect of alcohol on GSH efflux from liver cells to the blood, as explained above, but also because alcohol enhances the expression of the catalytic subunit of one of the enzymes participating in GSH synthesis in the liver, *viz. gamma*-glytamylcysteine synthetase (which is also called glutamate-cysteine ligase) [147]. This effect might perhaps outweigh the more acute inhibitory effect of alcohol on GSH synthesis and its effect on the rate of GSSG excretion to the bile as long as the alcohol consumption is not too high. It is not implausible that the stimulating effect of moderate doses of alcohol both on the expression of the catalytic subunit of *gamma*-glytamylcysteine synthetase and on the efflux of GSH from liver cells to the blood (which in combination would be expected to lead to elevation of the blood plasma GSH concentration, especially when the alcohol is consumed in connection with protein-rich hot meals) might in large measure explain the protective effect of moderate alcohol-consumption against coronary heart disease, metabolic syndrome and diabetes mellitus that appears now to be well documented through epidemiological studies [148-150].

### Prostaglandin biosynthesis, other lipid metabolites, pain and drugs

Several prostaglandins and other eicosanoids function as important sensitizers of C-fibres [90]. These are very thin (unmyelinated) peripheral nerve fibres comprising different subtypes with a number of different functions both in normal physiological regulation and during disease processes. Some of them are important mediators of nociceptive pain [151], but C-fibres can also secrete neuropeptides (such as tachykinins and CGRP) that are important mediators of inflammation [152-154].

#### Role of oxidized LA metabolites as agonists of the heat- and acid-sensitive vanilloid pain receptor

It has recently been reported that four oxidized metabolites of LA, *viz. *9- and 13-hydroxyoctadecadienoic acid (9- and 13-HODE), as well as 9- and 13-oxoODE, function as agonist ligands of the C-fibre vanilloid receptor (also called the capsaicin receptor) [155,156]. 9- and 13-HODE can be produced from LA by the action of various (12S)-lipoxygenase isozymes [157,158], followed by reduction of the hydroperoxides first formed by enzymes such as GPx-4 or GPx-1, depending on whether the LA hydroperoxides first formed are still bound to the membrane or not. 13-HODE can, moreover, also be formed by 15-lipoxygenase acting on LA [159], followed by GPx-catalysed reduction of the hydroperoxide formed in the 15-lipoxygenase reaction.

The vanilloid receptor is activated by low extracellular pH [160] and by noxiously high temperature (>43°C) [161]. It is also activated by capsaicin, which is the active substance in red pepper (*Capsicum *sp.) [162]. This presumably explains why foods rich in red pepper produce a burning sensation in the mouth and throat ('hot' foods). It has now been found that the activating effect of high temperature on the vanilloid receptor is caused by activation of enzymes converting phospholipid-bound LA into 9- and 13-HODE [156]. This presumably means that temperatures above 43°C must be associated with 12-lipoxygenase and/or 15-lipoxygenase activation (perhaps in large measure as a consequence of incipient cell death associated with lipoxygenase activation in the mitochondria).

It should be expected that the quantities of 9- and 13-HODE that will be formed at a certain temperature must depend on the concentration of LA in the membrane lipids, with a higher LA/oleic acid ratio in the membrane leading to enhancement of the quantities of 9- and 13-HODE that will be formed. The (12S)-lipoxygenase and 15-lipoxygenase have similar catalytic mechanisms as for the first step of the cyclooxygenase reaction, and they must therefore be activated by oxidation in a similar way as is needed also for COX. It has been reported that glutathione peroxidases (such as GPx-1 and GPx-4) counteract the activation of 15-lipoxygenase and 12-lipoxygenase [163-165], similarly as they counteract the activation of COX [77,163,164] and 5-lipoxygenase [166-168], with the rate of scavenging of the activator molecule (at a given concentration of the latter) being proportional to the concentration of the enzyme itself as well as to the second power of the GSH concentration. It can thus be expected theoretically that a high dietary LA/oleic acid ratio, low Se intake and poor GSH status may interact synergistically as causes of much 9- and 13-HODE production and hence much activation of the vanilloid receptor (leading again to more intense pain).

One should on this background not exclude the possibility that the high prevalence of chronic pain conditions found in the adult population in Norway may in part be explained as a side effect of the recommendations given by Norwegian health authorities (both to the general public and to the food industry) to increase the intake of LA in the general population by recommending that the LA concentration in margarine should be high and that people should consume LA-rich margarine and vegetable oils in preference to butter and other fat from animals other than fish. Thus, while it may be a matter of debate whether a high intake of LA has helped to reduce the incidence of ischemic heart disease or not, there is now strong reason to believe that the recommendations may have had an important (though wholly unintended) negative side effect by enhancing the severity (and most likely also the prevalence) of chronic pain problems in the adult population.

On the positive side, it should be noted that the 9-HODE [169,170], 13-HODE [170] and the 13-HODE metabolite 13-oxo-ODE [171] have been reported to function as agonist ligands of the PPAR-*gamma *receptor; however, it has also been reported that 13-(S)-HODE up-regulates the MAP kinase signaling pathway and subsequently down-regulates PPAR*gamma *in human colorectal carcinoma cells [172]. The same substance can thus have effects going in opposite directions, as far as PPAR*gamma *activation is concerned. Enhanced PPAR*gamma *stimulation would be expected to beneficial in various disease situations, not only in diabetes type 2 [82], but also in cardiac failure [173].

13-S-hydroxyoctadecadienoic acid has also been reported to down-regulate PPAR-*delta*, which can in turn lead to induction of apoptosis in colorectal cancer cells [174]. It may thus be concluded that oxidized LA metabolites can have favourable as well as unfavourable effects in various disease conditions, and it is not easy to find out, when talking about the health situation of the population as a whole with many different diseases (and perhaps not even for a single disease, such as cancer), whether the favourable or the unfavourable effects should be considered the most important ones.

#### Prostaglandin overproduction because of poor diet can lead to enhancement of pain and neurogenic inflammation because of C-fibre hypersensitivity

Prostaglandin (PG) overproduction because of poor diet (with too much AA and/or too little Se or too little GSH precursor amino acids) leads to sensitization of the C-fibres [90] to pain-inducing stimuli of all kinds (including low extracellular pH and high concentrations of 9- and 13-HODE). At the same time, it leads also to enhanced release of proinflammatory peptides from the C-fibres, which means intensification of almost any kind of inflammatory reaction in C-fibre-innervated tissues, including the skin, skeletal muscle, synovial membranes and the mucosa of the upper and lower airways.

In ischemic pain conditions (including pain caused by skeletal muscle spasms or static overload), one must expect that there will be a synergistic interaction between prostaglandin overproduction leading to C-fibre hypersensitivity and enhanced lactic acid production in the ischemic area, leading to enhanced acidic pH-mediated [160] activation of the vanilloid receptor. The rate of lactic acid production in a tissue area with limited oxygen supply is limited by the availability of carbohydrate precursors (glucose or glycogen) entering the glycolytic pathway. Since the quotient between rates of glucose and oxygen diffusion into the hypoxic area must be directly proportional to the blood sugar concentration, it must be expected that higher blood sugar concentrations will lead to more lactic acid production in ischemic or strongly hypoxic areas, which means stronger activation of the vanilloid receptors in these areas and hence more pain.

It must therefore be expected that there will be a synergistic interaction between high blood sugar levels (leading to enhanced vanilloid receptor activation) and prostaglandin overproduction (leading to C-fibre hypersensitivity) as causes of enhanced pain in all such common skeletomuscular disorders that are associated with a tendency for abnormal static loads or muscle spasms in the painful areas. A combination of overconsumption of AA, suboptimal Se intake, high consumption of high glycaemic load foods and little physical activity (which means less rapid consumption of blood sugar by the muscles) can not be favourable for the chronic pain patient, and one of the best ways to help him would probably be a life-style intervention where all these factors can simultaneously be corrected. Considering what is now the average composition of the diet in many of the western industrial countries (with a combination of very high AA intake, high consumption of high glycaemic load foods and often far from optimal Se intake), it should come as no surprise that the prevalence of chronic pain problems in the adult population of many of these countries is high.

#### Possible role of oxidative stress and impaired antioxidant defense as causes of PKC-mediated C-fibre hypersensitivity

C-fibres can also be sensitized by activation of protein kinase C (PKC) [90], and they contain several PKC isozymes that can be activated by oxidative stress [90]. It is therefore possible that Se or GSH depletion leading to impaired scavenging both of H_2_O_2_, organic hydroperoxides and peroxynitrite inside the C-fibres could be another important cause of C-fibre hypersensitivity, leading to enhancement of pain sensitivity and neurogenic inflammation. It is also possible that enhancement of the rate of reactive oxygen species (ROS) production inside C-fibre mitochondria because of mitochondrial DNA aging (natural or unnatural/pathologic, *e.g. *smoking-induced or drug-induced) could have a similar effect [90], with enhancement of mitochondrial ROS production and impairment of the capacity of ROS-scavenging enzymes interacting synergistically with each other as causes of PKC-mediated C-fibre hypersensitivity and hence more pain.

PKC is also activated by diacylglycerol (DAG) [82], and it is possible that factors such as hyperglycemia known to cause abnormal enhancement of the DAG concentration and therefore activating PKC and NAD(P)H oxidase in endothelial cells (as will be discussed later) also could play a role as contributory causes of hyperalgesia mediated by C-fibre PKC. It has already been mentioned that hyperglycemia also must be expected to lead to enhancement of lactic acid production in muscles under conditions of severely restricted oxygen supply, which will lead to enhanced stimulation of vanilloid receptors. A double bonus may thus be expected if the pain patient can change his diet and exercise more in order to keep the blood sugar level better under control.

The practical significance of mechanisms leading to C-fibre hypersensitivity is suggested by the fact that all conventional NSAIDs (non-steroidal anti-inflammatory drugs) function as cyclooxygenase inhibitors (even though this may not be their only mechanism of action) [82]. These drugs are very commonly used for treatment of acute as well as chronic diseases and injuries associated with pain and inflammation. Their analgesic effect can in large measure be explained as a result of reduced PG production, probably both peripherally (leading to less PG-induced sensitization of the C-fibres) and in the central nervous system. None of the NSAIDs are without side effects, which sometimes can be severe [82]. It should be noted that these drugs will most likely not help to correct the problem of C-fibre hypersensitivity caused by oxidative stress-mediated or DAG-mediated activation of PKC. One should therefore expect that they may have only limited effect in situations where PKC-mediated C-fibre hypersensitivity is an important part of the problem.

Dietary or other interventions for reducing oxidative activation of PKC isozymes in C-fibres (by optimizing the functions of C-fibre mitochondria and of their cellular system of antioxidant defense) must be expected to affect other cell types as well and lead to reduction of the synthesis of prostaglandins by leukocytes in the painful areas (for reasons that have already been explained above). A double bonus may thus be expected for such interventions, when prostaglandin-mediated and PKC-mediated C-fibre hypersensitivity can be reduced simultaneously by enhancing the intake of antioxidant nutrients or by using such hormones, such as melatonin, or dietary hormone precursors (such as tryptophan and vitamin D) that can help to enhance the expression of antioxidant enzymes and/or improve the function of dysfunctional mitochondria, at least in some cell types and organs [122].

In skeletal muscle ischemic pain, a triple bonus may be expected from the application of a suitable combination of antioxidant nutrients and hormones, since there is much research literature showing that several antioxidant nutrients (including Se, GSH, taurine and carnosine) and also melatonin at high dosage levels have substantial protective effects against damage caused by ischemia and reperfusion both in the heart and several other organs [154], including skeletal muscle (for some of the substances concerned, even though there are fewer experimental studies of this kind on skeletal muscle than on the heart, brain and liver). It goes far beyond the scope of the present article to survey and give references to all this literature, but much of it can easily be found on PubMed by combining the appropriate search words (*e.g.*: heart selenium ischemia protection).

While such methods for ameliorating skeletomuscular pains should not be regarded as substitutes for conventional physical therapy methods for treatment of the diseases concerned (including a combination of muscle relaxation therapy and physical exercise), there must be good reason to expect that the physical therapy methods, when effective, must synergize with dietary intervention or endocrine treatment methods either for correcting C-fibre hypersensitivity, for decreasing lactic acid production in the muscles under severe hypoxia, or for improving the function of the muscle cells under conditions of restricted oxygen supply and protecting them against damage caused by ischemia and subsequent reperfusion. A combination of both forms of therapy (thus trying to protect the painful muscle both from the inside and from the outside simultaneously) would be expected, especially for the more difficult cases, to give much better results than either applied alone. It must also be expected that a combination both of internal and external protection of vulnerable muscles must be better than either method alone for effective prophylaxis at a population level or for especially vulnerable groups (*e.g. *music students and musicians).

#### Mechanism of action and side effects of acetaminophen: is acetaminophen an important mitochondrial mutagen in the brain?

The mechanism and sites of action of another commonly used analgesic drug (which is not considered an NSAID because it does not have much antiinflammatory effect), *viz. *acetaminophen (paracetamol) has been subject of much controversy; it has been suggested that it might act by inhibition of a splice variant of COX-1 that has been called COX-3 and that is expressed in the central nervous system, but not peripherally [175]. More recently, evidence has been presented that suggests acetaminophen may function as a selective COX-2 inhibitor in humans [176].

It has earlier been proposed that acetaminophen can function as a cyclooxygenase inhibitor by reduction of a group at the active site of the enzyme that must be oxidized for activity [177,178]. In a study from 2001 it was found that reduced glutathione (GSH) and GPx enhanced the potency of acetaminophen as an inhibitor against both purified ovine COX-1 (oCOX-1) and human COX-2 (hCOX-2) approximately 30-fold to give IC_50 _values of 33 microM and 980 microM, respectively [178]. Acetaminophen was found to be a good reducing agent of both oCOX-1 and hCOX-2 [178]. These observations were thought to be consistent with a mechanism of inhibition in which acetaminophen acts to reduce the active oxidized form of the cyclooxygenase to the resting form [178]. Inhibition would therefore be more effective under conditions of low peroxide concentration (when activation of the enzyme by oxidation takes place more slowly), consistent with the known tissue selectivity of acetaminophen [178].

The poor anti-inflammatory effect of acetaminophen that has been observed in most, but not all [179] studies might then be explained as a result of the common tendency for inflammatory processes to be attended by high oxidative and sometimes also high nitrative stress (especially in rodent models of inflammation, since inducible NO synthase is expressed more easily in rodent leukocytes than in human leukocytes), which would lead to a high rate of COX activation by oxidation caused either by H_2_O_2 _[124,180], organic hydroperoxides [77,180] or peroxynitrite [125]. The reason why a better anti-inflammatory effect was found in Skjelbred and Løkken's study [179] could perhaps be that they used a model of inflammation (*viz. *the magnitude of the inflammatory reaction following tooth extraction) that conceivably might be associated with less oxidative stress than most other common forms of inflammation. More recent observations [181,182] seem to have confirmed the validity of Ouellet and Percival's observations [178], but a new mechanism of action for acetaminophen has also been discovered, *viz. *conversion into an active metabolite (*p*-aminophenol), which is conjugated with arachidonic acid by fatty acid amide hydrolase to form a compound called AM404, which exerts an analgesic effect through cannabinoid receptors [181].

Acetaminophen has been reported to be converted by cyclooxygenases both through 1- and 2-electron oxidation [177,183,184] into protein-reactive metabolites [185]. It is a plausible hypothesis that the acetaminophen metabolites that are formed by cyclooxygenases (especially the product(s) of 1-electron oxidation) can be covalently bound to DNA molecules as well and therefore might be mutagenic. It is thus possible that the same redox reactions that explain why acetaminophen can inhibit cyclooxygenases [178] also may convert this drug into DNA-reactive metabolites.

When health authorities all over the world started, many years ago, to recommend acetaminophen to be used, rather than acetylsalicylic acid (aspirin), as the first choice drug for treatment of common pain conditions and fever, one of the main reasons for this was the risk of development of Reyes' syndrome in children taking aspirin [82], while acetaminophen was without this side effect [82]. Another important reason was that the observed death rate because of acute side effects was clearly higher for aspirin (mainly because of severe gastric haemorrhage) than for acetaminophen. The recommendations to prefer acetaminophen rather than aspirin as the first choice drug for treatment of ordinary (not overly severe) pain and ordinary fever are understandable on background of what was known at the time when they first were issued (at which time the genotoxicity of acetaminophen still was unknown). But it is more difficult to understand why these recommendations were not changed after it had been reported, already some 25 years ago [186], that acetaminophen is mutagenic, which has later been confirmed by other groups [187,188]. It has also been reported later that acetaminophen interferes with DNA repair [189,190].

Acetaminophen itself does not react with DNA molecules, but while much of it is conjugated and excreted before oxidation [82], another part is oxidized by various forms of cytochrome P_450_, including cytochrome P_450_2E1 (CYP2E1). This process gives rise to the highly reactive and toxic metabolite *N*-acetyl-*p*-benzoquinone imine [82], which is presumably one of the most important mutagenic species formed when acetaminophen is metabolized in the liver or other organs. If the reaction mechanism of CYP2E1-catalyzed acetaminophen oxidation is similar as for other and better studied CYP2E1-catalyzed reactions, such as the CYP2E1-catalyzed oxidation of alcohol [141,191], one must also expect that CYP2E1-catalyzed oxidation of acetaminophen will be attended by formation of reactive oxygen species (ROS), peroxynitrite and perhaps organic radicals that also will be mutagenic.

*N*-acetyl-*p*-benzoquinone imine is detoxified by spontaneous reaction with reduced glutathione (GSH) [82]. One may then expect theoretically from diffusion and reaction kinetics that the steady-state concentration will decay quasi-exponentially as a function of distance from the sites of production, with the decay curve being steeper when the intracellular GSH concentration is high than when it is low. Cytochromes P_450 _are membrane-bound enzymes that are found especially in the endoplasmic reticulum and the inner mitochondrial membrane, even though they have been observed also in other parts of the cells, such as the outer nuclear membrane, different Golgi compartments, peroxisomes and the plasma membrane [192]. For brain cells, it has been reported that CYP2E1 is partly localized to the mitochondria [193,194].

The distance is evidently much shorter from the inner mitochondrial membrane to mitochondrial DNA than between the endoplasmic reticulum and nuclear DNA, at the same time as there are no other membranes hindering the passage of reactive acetaminophen metabolites between the inner mitochondrial membrane and the mitochondrial chromosomes (in contrast to the situation for nuclear DNA, where a double membrane hinders the diffusion of reactive acetaminophen metabolites from the cytosol into the nucleus). There are also no histones in the mitochondria, leaving the mitochondrial DNA much less protected against mutagenic molecules compared with much of the nuclear DNA (especially all such genes that are not actively expressed, and also so-called junk DNA), that is well shielded because of complex formation with histones and other protein molecules in combination with multiple DNA coiling. This coiling will sterically make it more difficult for mutagenic molecules coming from the outside to get access to the inner part of the coil. It is therefore good reason to suspect that acetaminophen may be far more mutagenic to mitochondrial than to nuclear DNA, at least in the brain. An important, perhaps not adequately studied question concerns the efficacy of repair mechanisms for acetaminophen-induced DNA lesions, other than oxidative DNA damage, in the mitochondria compared to the nucleus.

#### The connection between acetaminophen and asthma: is it a consequence of mutagenic effects in mitochondria in the lower respiratory tract?

More recently, it has been shown in epidemiological studies that the use of acetaminophen by children [195] or by their mothers during pregnancy [196,197] is associated with significantly enhanced risk of asthma or other respiratory symptoms - which perhaps might be a consequence of the mutagenic effects of this drug, especially for mitochondrial DNA, either in leukocyte progenitors, C-fibres or other cell types in the lower airways (such as smooth muscle cells or epithelial cells). If it does not happen as a direct consequence of adaptive immunity (with acetaminophen functioning as an allergen), which appears highly unlikely in this case, a mutagenic effect is by far the most plausible mechanism for explaining how the organism can "remember" exposure to a drug over several years to account for long-term (or permanent and irreversible) side effects.

Too much mutations in mitochondrial DNA must be expected to lead to enhancement of the rate of mitochondrial ROS production [90], which may in turn lead to enhanced expression not only of protective antioxidant genes, but also of a large number of proinflammatory genes that are positively regulated [198,199] by the redox-regulated [128,198,199] transcription factors NF-*kappa*B and AP-1. This can lead to enhanced expression of many of those proinflammatory genes that are important in asthma and other allergic diseases [199,200].

In the C-fibres, it must also be expected that enhanced ROS production will lead to activation of several isozymes of protein kinase C (PKC) that have been found in C-fibres and are activated by oxidative stress [90], at the same time as it is well documented that PKC activation leads to sensitization of the C-fibres [90]. One of the likely consequences of this will be enhanced secretion of proinflammatory peptides from C-fibres in the lower airways [154], while another possible consequence could be enhanced activity over a vagal reflex arc causing secretion of acetylcholine from parasympathetic nerve fibres in the lower airways. This, however, is a difficult and complicated topic since there is evidence for differential reflex regulation of cholinergic and noncholinergic parasympathetic nerves innervating the lower airways - with different reflex pathways associated with vagal nerves having opposite effects on the tone of bronchial smooth muscle cells [201].

It may on background of the observations linking acetaminophen both with mitochondrial DNA mutations and asthma be good reason to ask, what would be the more sensible strategy, either as now to correct pain conditions resulting from prostaglandin overproduction by highly liberal distribution of the mitochondrial mutagen acetaminophen even to children and young adults, or to restrict the dietary intake of AA while enhancing the intake of antioxidant nutrients such as GSH/sulphur amino acids and Se (but also taurine, carnosine/anserine and polyphenolic plant antioxidants) that hopefully might help to decrease cyclooxygenase activation and COX-2 expression? Is it science-based medicine when regulatory agencies and governments are reluctant to draw what would appear to be the only natural conclusions from all those reports that have shown that acetaminophen is mutagenic or that link it with enhanced risk of asthma, while neglecting all those even more numerous reports that show that it is harmful to eat too little EPA + DHA and too much AA?

### Prostaglandin biosynthesis, NSAIDs, COXIBs and cancer

COX-2 is expressed in many, but far from all tumour cell populations, being especially common in colon cancer [202]. But tumours (even in such cases when the tumour cells themselves don't express this enzyme) do also contain other COX-2-expressing cell types, including endothelial cells, where COX-2 is induced by the proangiogenic factor VEGF, released by tumour cells [203]. Tumour endothelial cells release COX-2-derived PGH_2_, which can be transformed into PGE_2 _by PGE synthase-expressing tumour cells [203]. PGH_2 _can also be converted into PGI_2 _by the endothelial cells and to TxA_2 _by platelets [82]. *In vivo *models of thromboxane A synthase or PGI synthase overexpression indicate proangiogenic effects for TxA_2 _and antiangiogenic effects for PGI_2 _[203]. Since PGH_2 _functions as an agonist ligand of the thromboxane A receptor (which is often called the thromboxane/prostaglandin endoperoxide receptor), one must expect that PGH_2 _itself will help to stimulate tumour angiogenesis, even without conversion into TxA_2 _or PGE_2_. COX-2 can, moreover, also be found in tumour-infiltrating leukocytes, such as macrophages, and may thus play an important role in tumour biology even in many of those cases where the tumour cells themselves don't express this enzyme.

Overproduction of PGE_2 _in tumour cell populations has a number of harmful effects, including stimulation of tumour angiogenesis [203-206], *i.e. *enhancing the ingrowth of blood vessels in the tumour, which will enable the tumour to grow faster by improving both its oxygen and nutrient supply (which normally are very important for limiting tumour growth [207]). It also suppresses the activities of various classes of leukocytes, such as NK cells [208-210], lymphokine-activated killer (LAK) cells [211] and CD8^+ ^T cells [212,213], that are important for tumour cell killing [208,209,214,215]. PGE_2 _can, moreover, also inhibit tumour cell apoptosis, at least in some tumour cell lines [216], if not necessarily in all types of tumour cells.

At the same time, it should not be forgotten that the above-mentioned anti-tumour immunological functions - that overlap strongly with NK-cell- and T-cell-mediated functions important for antiviral immunological defense - also depend on the glutamine, tryptophan, GSH and Se status of the patient [122,154,217]. As an example can be mentioned that NK cells normally will secrete the Th1-associated cytokine interferon-*gamma *following simultaneous stimulation with interleukin-12 (IL-12) and interleukin-2 (IL-2), provided that they are not GSH-depleted [217]. But GSH-depleted NK cells will instead secrete the Th2-associated (and Th1-inhibitory) cytokine interleukin-10 (IL-10) following double stimulation with IL-12 and IL-2 [217].

Epidemiological studies have shown that regular consumption of traditional over the counter nonsteroidal anti-inflammatory drugs (NSAIDs) or selective COX-2 inhibitors is associated with significant reduction of the death risk not only from colon cancer, but also from various other forms of cancer [218]. In a recent meta-analysis, it was found that regular intake of over the counter NSAIDs produced highly significant composite risk reductions of 43% for colon cancer, 25% for breast cancer, 28% for lung cancer, and 27% for prostate cancer [218]. Furthermore, it was found in a series of case control studies that daily use of a selective COX-2 inhibitor, either celecoxib or rofecoxib, significantly reduced the risk for each of these malignancies [218]. The evidence is now considered compelling that anti-inflammatory agents with selective or non-selective activity against cycloooxygenase-2 (COX-2) have strong potential for the chemoprevention of deaths from cancers of the colon, breast, prostate and lung [218].

The question can be raised to what extent the observed effect on death risk from cancer following use of either nonselective NSAIDs or COXIBs might be a consequence of primary chemoprevention because of an antimutagenic effect of COX-2 inhibition (*i.e. *inhibition of the appearance of the first malignant cells that later can give rise to a tumour cell population), and to what extent the death risk reduction might instead be explained as a consequence of delayed progression of the disease after it has started. From what is known about effects of prostaglandins in cancer, as well as about COX-2 itself (since COX-2 is an enzyme that can also oxidize other substrates than polyunsaturated fatty acids, and some of the products of such reactions could be mutagenic), it must be judged most reasonable to believe that the main effect could be on the rate of progression of the disease (because of poorer angiogenesis, higher tendency for apoptosis among the tumour cells and better antitumour immunity) rather than an antimutagenic effect leading to primary chemoprevention. If this conclusion is correct, it would obviously have important implications for the treatment of all such forms of cancer where COX-2 is expressed in the tumour cells (which should now be easy to find out from biopsy studies), since there is no reason to believe that the delaying effect of COX-2 on disease progression should be restricted to the earliest stages of development of the disease.

### Can a better diet enhance the therapeutic effect while reducing the risk of adverse side effects for COXIBs during therapy of rheumatoid arthritis and cancer?

It is probably much better to limit the production of prostaglandins in a tumour cell population by a combination both of dietary and pharmacological intervention rather than by pharmacological intervention alone, since with such combinations, it should be possible to obtain the same proportional reduction of prostaglandin biosynthesis in the tumours with much less side effects than with pharmacological intervention alone. With less side effects, it is also possible to enhance the intensity of treatment (*i.e. *aim for a larger proportional reduction of PG synthesis inside the tumour than one could have done with pharmacological therapy alone). It should therefore be considered mandatory for all cancer patients, especially in such cases where the tumour cells express COX-2 or COX-1, to restrict the dietary intake of AA, at the same time as the total intake of other unsaturated fatty acids (especially *omega*-3 fatty acids and oleic acid) that compete with AA for incorporation in the same positions in the membrane lipids of the tumour cells should be increased. Given the important role of COX-2 expression also in other cell types in the tumours, such as endothelial cells [203] and macrophages, these guidelines may be expected to be therapeutically useful even in a majority of such cases when the tumour cells themselves don't express either COX-2 or COX-1.

The patients should, moreover, also be advised to enhance the dietary intake of such antioxidant nutrients (*e.g. *Se and GSH, or GSH precursors, but also such plant oxidants that have been reported to have similar effects) that hopefully may help to reduce COX-2 expression and activation in the tumour cell population, tumour-associated macrophages and tumour endothelial cells. These antioxidant nutrients can in any case (even if they should not be effective for reducing prostaglandin synthesis in the tumour cells) not be expected to have any harmful side effects, but may rather help to improve the overall health and quality of life for the patients, *e.g. *by helping to maintain muscular strength, reduce the risk of cardiovascular complications [122], and strenghten immunological functions [122,154,217].

Observations confirming that COX-2 blockade is effective for cancer prevention have been tempered by observations that some selective COX-2 inhibitors pose a risk to the cardiovascular system [218]. Nevertheless, a meta-analysis of independent estimates from 72 studies provides no evidence that the selective COX-2 inhibitor, celecoxib, influences the relative risk of cardiovascular disease (composite relative risk = 0.98, 95% CI = 0.88-1.10) [218].

It is commonly assumed that the main reason why traditional, non-selective NSAIDs can have sometimes severe gastrointestinal side effects is inhibition of COX-1 in the stomach [83]. One might then ask the question: why does not the same happen - when the rate of EPA oxidation by COX-1 is only 10% of the rate of AA oxidation by the same enzyme [76] - also when one reduces eicosanoid biosynthesis via COX-1 by reducing the AA/(EPA + DHA) ratio of the diet? One possible answer could be much higher local drug concentration (because of local absorption) in the gastric mucosa following ingestion of the drug (but also in the mucosa of the upper intestine), compared to the rest of the body following absorption in the intestine (and transport away from the intestine by the blood). Something similar can not happen following ingestion of phospholipids and triglycerides containing long-chain polyunsaturated fatty acids.

Selective COX-2 inhibitors (COXIBs) have been shown to possess much improved gastrointestinal tolerability with reduction of the incidence and/or severity of gastrointestinal adverse events, when compared with nonselective inhibitors (that also inhibit COX-1) [83]. An unexpected cardiovascular toxicity did, however, emerge during COXIBs post marketing outcome studies [83]. This COXIB-associated cardiovascular toxicity has multiple manifestations, which include the induction of myocardial infarction, oedema, thrombosis, blood pressure destabilization and death [83]. It has led to withdrawal from the market of two of the drugs concerned, *viz. *rofecoxib and valdecoxib, while celecoxib is still in the market because the risk of cardiovascular side effects of this drug is significantly less than for those that have been retracted [83].

It has been thought that the cardiovascular side effects of COXIBs may in large measure be explained as a result of COX-2 inhibition in endothelial cells, leading to a disturbance of the balance between prostacyclin synthesis in the endothelial cells and thromboxane synthesis in the platelets [81]. The thromboxanes (TxA_2 _and TxA_3_) are potent platelet aggregators and vasoconstrictors [82], while the prostacyclins (PGI_2 _and PGI_3_) are potent anti-aggregators and vasodilators [81,82]. Although COX-2, in contrast to COX-1, has often been regarded as an inducible enzyme that only has a role in pathophysiological processes like pain and inflammation, experimental and clinical studies have shown that COX-2 is constitutively expressed in some tissues like the kidney and also in vascular endothelium, where it executes important physiological functions and is needed for the maintenance of vascular integrity [81]. Prostacyclin is formed to a significant extent by COX-2, and its levels are reduced to less than half of normal when COX-2 is inhibited by COXIBs [81].

But the prostacyclin/thromboxane balance is also heavily influenced by the dietary AA/(EPA + DPA + DHA) ratio. A high dietary AA/(EPA + DPA + DHA) ratio enhances the risk of thrombotic events, while a low dietary AA/(EPA + DPA + DHA) ratio has the opposite effect, as first shown by the studies of Dyerberg and collaborators on Inuits in Greenland [219]. This was earlier explained by the assumption, now shown to be false [76] that TxA_3 _(that is formed from EPA) is inactive, whereas the prostacyclin PGI_3 _(that is also formed from EPA) is fully active. Now another explanation must be sought instead of the false assumption that TxA_3 _is inactive. Part of this new and hopefully more correct explanation can probably be found in the different substrate specificities for COX-1 compared with COX-2, with the rate of AA conversion to PGH_2 _by COX-1 being 10 times higher than the rate of EPA conversion to PGH_3 _by the same enzyme, whereas the difference between the rates of oxidation of AA and EPA by COX-2 is much smaller [76]. Enhancement of the dietary EPA/AA ratio will therefore affect the rate of prostacyclin synthesis in the endothelium less than it affects the rate of thromboxane synthesis in the platelets. Also, when EPA is a better inhibitor of AA oxidation by COX-1 than for AA oxidation by COX-2 [76], this means that it will inhibit TxA_2 _synthesis in the platelets more than it inhibits PGI_2 _synthesis in the endothelium - which is another mechanism acting in the same direction. DHA, with 22 carbon atoms and 6 double bonds, is not a precursor for prostaglandin or thromboxane biosynthesis. But it functions as a competitive inhibitor for the oxidation of polyunsaturated fatty acids with 20 carbon atoms in the platelet cyclooxygenase reaction and therefore as an inhibitor of the biosynthesis of thromboxane A_2 _[220].

Docosapentaenoic acid (DPA) has been reported to function as a potent inhibitor of platelet aggregation [220]. This can most likely be explained as a consequence of the same effect that EPA and DHA also have as inhibitors of platelet aggregation caused by TxA_2 _or stable TxA_2 _analogs because they bind to the platelet thromboxane A_2_/endoperoxide receptor, where they function as blocking agents [221-223]. DHA was found to be more potent than EPA in blocking platelet aggregation induced by the stable thromboxane A_2 _mimetic, U46619 [221]. Prostaglandin endoperoxides, which are very short-lived, can also function as agonists at the receptor for thromboxane A_2_, which is therefore called the thromboxane A_2_/prostaglandin H_2 _receptor [221] or the prostaglandin-endoperoxide/thromboxane A_2 _receptor [223]. When AA is metabolized much faster than EPA by platelet cyclooxygenase, this will, of course, not only lead to faster synthesis of thromboxanes, but to faster synthesis of PGH as well.

But in tumours where the tumour cells release much VEGF leading to enhanced expression of COX-2 in the endothelium and enhanced release of PGH from endothelial cells [203], one must expect that a reduction of the intake of AA while enhancing the intakes of EPA and DHA will also help to reduce the release of PGH_2 _and of total PGH from the tumour endothelial cells (even though the effect of reducing the intake of AA and enhancing the intakes of EPA and DHA is not equally large for COX-2-mediated synthesis of PGH_2 _in the endothelium as it is for TxA_2 _synthesis in the platelets). At the same time it must be expected that EPA and DHA will help to suppress the proangiogenic effect that must be expected to occur for PGH_2 _coming from the endothelium, similarly as for TxA_2 _coming from the platelets [203], since PGH_2 _is an agonist ligand of the thromboxane A_2_/prostaglandin H_2 _receptor [221], while EPA and DHA function as blockers of this receptor [221-223]. Reducing VEGF-mediated angiogenesis by decreasing the intake of AA and enhancing the intakes of EPA and DHA is a principle that could presumably be useful also for the therapy of other diseases, where overproduction of VEGF is an important part of the pathogenetic mechanism (such as the neovascularisation of eye tissues that may happen as a complication of diabetes).

The rate of prostacyclin synthesis in the endothelium depends not only on the dietary AA/(EPA + DHA) ratio, but is also strongly influenced by the rate of reactive oxygen species (ROS), peroxynitrite and PUFA hydroperoxide production in the endothelial cells, as well as by the capacity of enzymes scavenging superoxide anion radical, organic hydroperoxides and peroxynitrite. This is because prostacyclin is irreversibly inhibited by low concentrations of peroxynitrite [224] and certain products of lipid peroxidation, including oxidized LDL [225,226].

Inhibition of prostacyclin synthetase in the endothelial cells must, however, be expected to have doubly harmful effect because it will not only lead to reduced synthesis of prostacyclin, but also to enhancement of the rate of PGH release from the endothelial cells when PGH is not converted at a normal rate to prostacyclin - with PGH_2 _functioning as a prostacyclin antagonist because it is an agonist ligand of the thromboxane A_2_/prostaglandin H_2 _receptor [221]. It is therefore plausible to speculate that the substantial VEGF-induced release of PGH_2 _that has been observed in tumour endothelial cells [203] may happen not only as a consequence of VEGF induction of COX-2 [203], but also because prostacyclin synthetase in the endothelial cells is simultaneously inhibited. It might furthermore be speculated that this might happen not only because of the effect of VEGF itself on rates of superoxide anion radical and peroxynitrite production in the endothelial cells, but also because of other sources of superoxide anion radical that can be converted into peroxynitrite in the tumour microenvironment, which might include not only activated phagocytes, but in a number of cases also the tumour cells themselves.

Peroxynitrite is formed in a very fast reaction between NO and superoxide anion radical [227]. The rate of peroxynitrite formation in the endothelium will thus be enhanced when the rate of production of superoxide anion radical - from endothelial NAD(P)H oxidase [228-230], by decoupling of endothelial NO synthase [231] and by leakage of electrons from the respiratory chain in the mitochondria [230,232] - is high. One must, moreover, also expect that the rate of peroxynitrite formation in the endothelium will be enhanced when the activity of one or both superoxide dismutases (one Cu/Zn-dependent and one Mn-dependent) in the endothelial cells is depressed, as can happen, at least in other cell types, as a consequence of copper deficiency [233] or manganese deficiency [234].

The rate of superoxide anion radical production by endothelial NAD(P)H oxidase can be enhanced *i.a. *by hyperglycaemia [228,229], by advanced glycation end products (AGEs) [230], by free fatty acids [228], and by angiotensin II [235], while the rate of superoxide anion radical production in the mitochondria of endothelial cells is enhanced *i.a. *by AGEs [230], by TNF-*alpha *[232], and very likely also by mitochondrial DNA aging (*i.e. *the progressive accumulation of mitochondrial DNA mutations as a function of age), similarly as is known for other cell types [90,236]. The effect of hyperglycemia on superoxide anion radical production by the endothelial NAD(P)H oxidase is mediated by enhanced production of diacylglycerol (DAG) in the endothelial cells, with DAG activating PKC [228]. It might be speculated, as earlier mentioned, that a similar hyperglycemia-induced enhancement of DAG synthesis also could play a role as one of the causes of hyperalgesia when it occurs in C-fibres.

Endothelial NO synthase uncoupling happens as a consequence of undersaturation of the enzyme with the cofactor 5,6,7,8-tetrahydrobiopterin [231], which can in turn happen as a consequence of too rapid oxidative degradation of this cofactor and accumulation of its oxidation product 7,8-dihydrobiopterin [231]. Endothelial dysfunction happening at least in part as a consequence of uncoupling of endothelial NO synthase is a very common complication of several different conditions that are associated with enhanced oxidative stress and/or impaired antioxidant defence in blood plasma and/or in the endothelium, including diabetes [237-239], hypertension [238], hypercholesterolemia [238], and chronic smoking [238].

It can be seen that there is a surprising degree of overlap between the etiopathogenesis of cardiovascular diseases and pathophysiological processes controlling the rate of tumour angiogenesis in cancer patients (by inhibiting the synthesis of antioangiogenic prostacyclin and enhancing the release of proangiogenic prostaglandin endoperoxides from tumour endothelial cells). Many of the same therapeutic interventions that may be beneficial for patients with cardiovascular diseases might hence be useful also for patients suffering from cancer.

Release of NO from tumour cells expressing inducible NO synthase (NOS-2) can also be expected to play a role in this context by contributing to enhancement of the rate of peroxynitrite production both in blood plasma and inside the endothelial cells, especially when the production of superoxide anion radical from various sources is high. When this leads to enhanced uncoupling of endothelial NO synthase (because of enhanced destruction of 5,6,7,8-tetrahydrobiopterin by peroxynitrite), it means that there will be more production of superoxide anion radical inside the endothelial cells, with the combination of NO coming from the tumour cells and superoxide anion radical coming from uncoupled endothelial NO synthase being a very effective method for inhibiting prostacyclin synthetase by peroxynitrite, so that the production of antiangiogenic [203] prostacyclin will be reduced at the same time as endothelial cell release of proangiogenic [203] PGH_2 _is enhanced. It is not unreasonable to speculate that this mechanism of stimulating tumour angiogenesis might be one of the most important reasons why expression of NO synthase-2 can give tumour cells a Darwinian fitness advantage, and why it can often be difficult to treat tumours expressing this enzyme (even though NO overproduction has multiple effects both harmful and beneficial for the tumour cells, with the net effect apparently being different in different forms of cancer). It might be well worth to test the effect of 5,6,7,8-tetrahydrobiopterin supplementation (in an attempt to normalize the function of uncoupled endothelial NO synthase [237,239]) as part of the therapy, *e.g. *in colon cancer when the tumour cells express NOS-2.

Peroxynitrite and water-soluble organic hydroperoxides are scavenged by various enzymes including the selenoproteins GPx-1 [126,127] and selenoprotein P, and also some of the peroxiredoxins [227,240,241]. GPx-1 uses reduced glutathione (GSH) as its reducing cofactor, displaying *tert-uni *ping pong kinetics [126]. For a given concentration of the oxidizing substrate (*e.g. *H_2_O_2 _or peroxynitrite), the rate of scavenging of the oxidizing substrate will thus be determined by the product of the concentration of the enzyme (which depends on Se status) and the second power of the GSH concentration.

The combined effect of all the above-mentioned factors that can influence the balance between PGH release from endothelial cells plus thromboxane production in the platelets on one side and prostacyclin production in the endothelium on the other side is most likely much higher, especially if several of these factors act simultaneously in the same direction, than that of selective COX-2 inhibitors at recommended dosage levels, even when compared to those that have now been retracted from the market. The conclusion can be drawn that it most likely may be harmful to use selective COX-2 inhibitors (especially those that have now been retracted from the market) if nothing is done to correct such other factors that may contribute to dangerous imbalance of the ratio of thromboxane production in the platelets to prostacyclin production in the endothelial cells. But it should most likely not be difficult to use dietary therapy (by simultaneously reducing the dietary AA/(EPA + DHA) ratio, improving the capacity of antioxidant enzymes in blood plasma and the endothelium and correcting NO synthase uncoupling) for decreasing to a very significant extent the cardiovascular risk associated with COXIBs, even the most dangerous ones. Such dietary interventions must at the same time also be expected to reduce the proangiogenic effect of VEGF released from tumour cells because they may help to reduce the rate of VEGF-induced release [203] of PGH_2 _from tumour endothelial cells (and in the case of EPA and DHA reduce its proangiogenic effect by blocking the thromboxane/prostaglandin endoperoxide receptor) while in the case of antioxidant nutrients also enhancing the production of antiangiogenic prostacyclin.

At the same time, it is important enough to be repeated that some of the dietary interventions that can be used for improving the thromboxane/prostacyclin balance will synergize with the COXIBs as causes of reduced prostaglandin production both in COX-2-expressing tumour cells, tumour-infiltrating [207] macrophages and tumour endothelial cells. The therapeutic ratio for the COXIBs can thus be improved because of simultaneous improvement of the therapeutic effect and reduction of the risk of harmful side effects, compared to the use of similar doses of COXIBs alone without simultaneous dietary intervention. This must be expected to be the same both when COXIBs are used for cancer therapy and when they are used for treatment of non-infectious inflammatory diseases, such as rheumatoid arthritis, psoriatic arthritis or Bekhterev's disease (ankylosing spondylitis).

For highly lethal diseases such as cancer, one should, of course, compare the risk of lethal cardiovascular side effects of a drug with the protection against dying from the disease itself (or from common complications to it) that the same drug can give. With a combination of optimal dietary intervention and COXIBs, it is reasonable to expect that the risk of dying from cardiovascular side effects of the drug (measured as the statistically expected average shortening of survival) will be almost negligible compared with the therapeutic benefit, considering the statistically expected average prolongation of the time of survival before the patient will die from his cancer. It is possible that this might apply even to those COXIBs that now have been retracted from the market, even though it might be more prudent to avoid them.

Given the role of COX-2 expression not only in tumour cells (in many, but far from all cancer patients), but also in tumour endothelial cells [203], as well as in tumour-infiltrating leukocytes, it would be expected that the proposed combination of dietary intervention with COXIBs might be therapeutically useful for a vast majority of cancer patients, and not only for such cases where the tumour cells express COX-2, although it might be especially important for them. This might even more be the case if this form of combined dietary/pharmacological therapy is combined with such dietary or pharmacological interventions that can help to reduce the production or reduce the effect (by using drugs to block the relevant receptors) also of substances other than PGE_2 _(such as lactic acid, adenosine and TGF-*beta*) that are commonly produced by tumour cells as anti-immunological defense weapons. Even better results might presumably be obtained by combining multifactorial therapies for suppressing tumour angiogenesis and tumor antiimmunological defense with such therapies that aim at specific or non-specific stimulation of antitumour immunological defense, *e.g. *using tumour vaccines or such hormones (*e.g. *melatonin at high dosage levels) that help to stimulate those parts of the immune system that are especially important for antiviral [90,154], antituberculosis and anticancer immunological defense.

It may be important to recognize that there is very broad overlap between the immunological defense methods used against viral infections, against infections with bacteria living intracellularly (*e.g. *tuberculosis) and against cancer, with NK cells and cytotoxic T lymphocytes being important in all these cases. It is an important consequence of this that there can be substantial overlap between antiimmunological defense methods used by tumour cells and those used by some of the bacterial pathogens, with tuberculosis (TB) probably being the best example (especially when considering the use of lactic acid as an antiimmunological defense weapon both in TB and cancer). It is, moreover, also much overlap comparing those metabolic changes that always happen as a consequence of infectious disease [135] and those happening in many cancer patients. These metabolic disturbances can lead not only to protein-energy malnutrition [135] (*e.g. *in AIDS and tuberculosis patients, and in form of cancer cachexia [133,134]), but also to various specific forms of malnutrition [135] (*e.g. *tryptophan, sulphur amino acid and GSH depletion [122,131,132]), leading in turn to further depression of antiviral [90,122,154,217], antibacterial [135,217] and antitumour immunological defense. At the same time it must also be expected that depletion of GSH or other antioxidant nutrients in the malnourished patient will lead to enhancement of COX-2 expression and the rate of prostacyclin synthetase inactivation in tumour endothelial cells, which means enhancement of the rate of PGH_2 _release from the latter [203] and more PGH_2_-induced and PGE_2_-induced (when the tumor cells express PGE synthase [203]) stimulation of tumour angiogenesis. All this means that much of the clinical experience that has been obtained with non-specific methods of immunostimulation for treatment of cancer patients (*e.g. *using high-dose melatonin, or agonists of Toll-like receptors) and regarding the role of nutrition in cancer patients also might be relevant for treatment of severe infectious diseases, such as tuberculosis, AIDS and hypervirulent avian influenza [154], and vice versa. Similarly, one must also expect that much of the knowledge obtained from animal experiments, clinical observations and epidemiological ones about the importance of nutrition factors and hormones for morbidity and lethality in viral and bacterial infections [90,135,154] may be highly relevant in cancer therapy as well. Better contact and collaboration than is common today in the fields of clinical nutrition and immunotherapy between clinical scientists working with cancer and those working with serious infectious diseases, such as tuberculosis and AIDS, could probably be most useful for both partners as well as for their patients.

### Epidemiological data concerning health benefits of fish consumption and/or high intake of long-chain *omega*-3 fatty acids

Several epidemiological and other studies suggest (even though the data are not completely consistent and unequivocal) that fish and also fish oil consumption in form of dietary supplements have marked protective effects against cardiovascular deaths, especially in form of sudden cardiac death (that may often happen as a consequence of malignant arrhythmia) [242-244]. However, interpretation both of the epidemiological data and the clinical intervention studies is fraught with problems, since marine fish does not only contain EPA and DHA, but also biodegradation-resistant organic environmental pollutants and toxic heavy metals (especially mercury) [51,245,246], as well as several other nutrients that either are known for certain to be cardioprotective or hypothetically might be so, such as Se [51,247-250], GSH and GSH precursor amino acids [251,252], anserine [253-255], vitamin B_12 _[256] and taurine [9-12,257] - and perhaps even arsenic (As) [51] in form of non-toxic organic compounds [258], since it has been reported that experimental As deficiency in animals can cause development of a cardiomyopathy histologically very similar to cardiomyopathy caused by Se deficiency [258].

Epidemiological data suggest also that the method of food preparation plays an important role, with some types of fried fish apparently being harmful [259,260], most likely because of production of toxic substances during food preparation either as a result of oxidation or because of exposure to high temperature. For intervention studies using fish oil capsules, one can not always be certain about the quality of the product that was used, *i.e. *whether the fish oil could have been oxidized or not. It would not be surprising if oxidized fish oil might have completely different physiological, pharmacological or toxic effects compared to fish oil as such.

A preliminary conclusion may be drawn from some of the studies concerned that EPA and DHA can protect against sudden cardiac death because they have a marked antiarrhythmic effect and therefore can protect against malignant arrhythmia [242,244,261]. It is possible that this effect is exerted mainly by EPA and DHA in form of free fatty acids rather than as eicosanoid metabolites, most likely as a consequence of binding to calcium and sodium channels in the cardiomyocytes [262,263].

### Is it possible to change poultry and pork meat to become more health-promoting than now?

It has been proposed that the combined effect of dietary intake of EPA and DHA and a number of other factors determining levels of EPA and DHA in an individual can best be assessed as the *omega*-3 index, defined as the sum of relative concentrations of EPA and DHA in red cells, and analyzed in a standardized fashion [69]. A very recently published review of the literature, expanded by measurements of the *omega*-3 index, indicates that the risk of sudden cardiac death correlates inversely with the *omega*-3 index [69]. For persons with an *omega*-3 index <4%, the risk is tenfold higher, as compared to persons with an *omeg*a-3 index >8% [69]. If this conclusion is valid, it is difficult to see that there can be any method more effective - and more cost-effective - for reducing the rate of sudden cardiac death in entire populations (*e.g. *in all of the United States, or in all of Russia), other than imposing new regulatory requirements both on animal feed producers and the farmers, making it mandatory that meat products, offal and eggs shall not have an AA/EPA + DHA) concentration ratio higher than, say, 2/1.

#### Optimizing the omega-6/omega-3 ratio and Se concentration of meat and eggs is better from an ecological point of view than encouraging enhanced global consumption of already overexploited fish resources

We think that all chicken meat as well as pork meat and eggs available for human consumption should have a favourable ratio between *omega*-6 and *omega*-3 fatty acids, especially when considering the long-chain ones such as AA, EPA and DHA. An important reason for this is that the currently exploited sources of the long-chain *omega*-3 fatty acids EPA, DPA and DHA are limited because of ecological limitations on total fish production in the sea and a tendency for overexploitation of many of the commercially most important fish stocks [264]. Every step towards increasing the concentration of these fatty acids in the regular human diet from sources other than fish may therefore be of importance, if it shall be possible to stop current overfishing and prevent that important fish stocks or even species (*e.g. *among tunas and sharks) shall go extinct in the near future, as may perhaps already have happened with that subpopulation (or subspecies) of cod that used to live on the Newfoundland Banks.

Another important reason for not encouraging the populations in Western Europe and North America to eat more fish than they already do is that fish traditionally has been very important for improving the nutritional quality of the diet for people living in many parts of Asia (such as Bangladesh) and Africa, not only as a source of protein and essential amino acids (such as lysine, tryptophan and the sulphur amino acids), but also (especially when whole fish products like dried kapenta are eaten) for nutrients such as calcium, iron, zinc, Se, iodine, long-chain *omega*-3 fatty acids, nucleotides and vitamin B_12 _[265]. But this important resource is now becoming increasingly scarce (*i.a. *as a consequence of local human population growth, but also as a result of other factors, such as heavy local pollution, overfishing, or diversion for the export market of fish resources that earlier were consumed locally), especially for the poorer part of the population living in those countries. And when global resources of wild fish are limited, it would be better if as much as possible of this limited resource could go to those who have the largest nutritional requirements because they can not afford to eat other animal foods, rather than to the rich man's table.

Fish is also one of the best dietary sources of Se [51], but it is neither difficult nor expensive to modify the feed composition of domestic animals living on land, so that the Se/protein concentration ratio in meat, offal, milk and eggs can be made equally as high as for fish products. The concentration of Se in wheat grown in Norway is low (wheat grown at Norwegian University of Life Sciences, Ås, Norway contains less than 20 microgram Se/kg wheat, own results), mainly as a consequence of poor bioavailability of soil Se for uptake in the plant roots. The commercial chicken feed concentrate is therefore supplemented with Se to avoid Se deficiency in the animals, which is given in form of inorganic Se (sodium selenite). There are dual benefits from the Se supplementation: improved health and performance of the animals and improved product quality for human consumption, with increased Se concentration and decreased drip loss during meat storage [266]. However, much of the Se added to the feed concentrate as sodium selenite is excreted and not incorporated into the meat. Supplementation of the feed with Se-enriched yeast or selenomethionine is much more efficient if the intention is not only to prevent Se deficiency diseases in the animals, but also to increase the Se concentration in meat for the benefit of the human consumer [20,267,268].

As Se is a very scarce trace element on our planet and total resources of commercially exploitable sulphide ores containing Se are limited, the Se concentration in the human diet should be increased in an efficient and sustainable way with as little waste as possible [268]. Se supplementation regimes in feed should therefore be evaluated for sustainability, and organic Se forms that may help to reduce overall waste should be preferred [268].

#### Could specifically tailored "functional food" meat be made for use in cancer therapy?

Meat products containing a favourable ratio between *omega*-6 and *omega*-3 fatty acids, an AA concentration considerably less than now and high Se levels might be desirable both for prophylactic reasons (in order to reduce morbidity and mortality from several important diseases in the general population) and for making it practically more easy to optimize the composition of the total diet for patients suffering from serious diseases such as cancer, coronary heart disease, rheumatoid arthritis, tuberculosis or HIV disease. For some of the patient groups concerned (*e.g. *patients suffering from colorectal cancer or rheumatoid arthritis), it would probably be useful to make specifically tailored functional food products that have especially high Se concentrations and AA as low as possible.

It has recently been reported that milk from cows fed diets enriched with Se-enriched yeast may have a more beneficial impact on bowel cancer compared to other Se supplements [269]. It might be speculated that this did not happen because Se from milk is more bioavailable than Se from yeast, but because of a synergistic interaction between Se and other antioxidant nutrients also found at high concentrations in the milk, such as oleic acid and sulphur amino acids. More specifically, it might be speculated that one of the mechanisms involved could have been a combination of oleic acid-induced suppression of superoxide anion radical in the endothelial cells [73], leading to reduction of the production of peroxynitrite, and improved scavenging of peroxynitrite by glutathione peroxidase [127] in the endothelial cells because their GSH concentration was enhanced. The combination of less rapid production and more rapid scavenging of peroxynitrite would have led to reduction of peroxynitrite-mediated inhibition of prostacyclin synthetase [224], leading in turn to reduction in the release from endothelial cells of proangiogenic [203] PGH_2 _and enhanced release of antiangiogenic [203] PGI_2_. But if this explanation for the experimental observations concerned should indeed be the correct one, something similar would also be expected when we use Se-rich, oleic acid-rich and AA-poor chicken meat as a source of Se for cancer patients, rather than only using Se pills.

This will, of course, be especially important in patients in whom the rate of sulphur amino acid catabolism is enhanced because they suffer from protein catabolic conditions, as has been demonstrated both in cancer and HIV disease [131,132]. This seems to happen mainly as a result of enhanced degradation of sulphur amino acids to sulphuric acid in skeletal muscle [132], very likely as a result either of cytokines (such as TNF-*alpha*) [90,122], proteolysis-inducing factor (PIF) coming from tumour cells [134], or other signal substances enhancing the rate of reactive oxygen species (ROS) production in the muscle cells. Enhancement of the rates of superoxide anion radical, peroxynitrite and H_2_O_2 _production in the muscle cells may conceivably lead to enhancement of the rate of irreversible hyperoxidation of sulphur atoms in cysteyl groups in protein and glutathione and perhaps also of sulphur atoms in protein methionyl groups (*i.e. *oxidation of the sulphur atoms in thiol or methionyl groups to an oxidation number where the oxidative lesion can no longer be repaired by enzymes such as thioltransferase, thioredoxin and methionine sulfoxide reductases).

It must be expected that many cancer patients, especially among those suffering from cancer cachexia (which may affect up to 50% of cancer patients [133]), will be depleted in GSH, which next must be expected to lead not only to enhancement of the total rate of prostaglandin biosynthesis for reasons that have earlier been explained, but also to enhanced peroxynitrite-mediated inhibition of prostacyclin synthetase and enhanced release of proangiogenic PGH_2 _from tumour endothelial cells. This must in turn be expected to lead to enhanced stimulation of tumour angiogenesis by PGH_2 _and PGE_2 _(and hence faster tumour growth), enhanced suppression of leukocytes important for antitumour immunological defense (such as NK cells, LAK cells and cytotoxic CD8^+ ^cells) by PGE_2_, and more eicosanoid-induced pain at the same time as there might conceivably also be exacerbation of pain as a consequence of more oxidative activation of PKC isozymes in the C-fibres [90]. At the same time, it can also be expected that GSH depletion will change the pattern of cytokine secretion from NK cells and other cell types towards reduction of the secretion of Th1-associated cytokines, such as IL-12 and interferon-*gamma *[154,217], and more secretion of Th2-associated cytokines, such as IL-10 [217]. But changing the Th1/Th2 balance in favour of Th2 (T-helper 2) will also mean suppression of antitumour immunological functions mediated by NK cells and cytotoxic CD8^+ ^T-lymphocytes.

For optimizing the diet of cancer patients (or other patients who suffer from protein catabolic conditions) who often also suffer from the problem that their appetite may be depressed (either because of the disease, or as a side effect of rough therapy with cytotoxic drugs), it will obviously be an advantage for the dietician and nurses to have access to a variety of different foods that all have a composition that is good for the patients. It might be easier to encourage the patient to eat as much nutritious food as he or she needs if the patient can himself decide whether he prefers fish, poultry meat or pork for dinner that day, while the dietician knows that they all have a composition that is good for the patient (without too much AA and with high Se concentration). The same will, of course, also be true for all other hospital patients as well as for all geriatric patients in nursing homes, whenever there is a problem with poor appetite or enhanced protein catabolism.

The importance of Se for cancer patients is supported by heavy theoretical arguments based on what is known about the biochemical functions of Se-dependent enzymes and the importance of Se not only for antioxidant defence and control of eicosanoid biosynthesis, but also for immunological functions [122,270]. But it is also consistent with the results of a clinical trial where patients who had earlier been treated for basal cell or squamous cell carcinomas of the skin were given 200 microg of Se per day or placebo to see if it could prevent new skin cancer [271]. The patients were treated for a mean of 4.5 years (SD 2.8 years) and had an average total follow-up of 6.4 years (SD 2.0 years) [271]. No effect on the recurrence of skin cancers was found, but analysis of secondary end points revealed that, compared with controls, patients treated with Se had a nonsignificant reduction in all-cause mortality (108 deaths in the Se group and 129 deaths in the control group [RR; 0.83; 95% CI, 0.63-1.08]) and significant reductions in total cancer mortality (29 deaths in the Se treatment group and 57 deaths in controls [RR, 0.50; 95% CI, 0.31-0.80]), total cancer incidence (77 cancers in the Se group and 119 in controls [RR, 0.63; 95% CI, 0.47-0.85]), and incidences of lung, colorectal, and prostate cancers [271].

Given the comparatively short duration of this experiment, compared to what is known about the latency period *e.g. *for lung cancer among smokers, it is difficult to explain the observations from this experiment (if valid) as caused only by a primary prophylactic effect. It is more plausible to explain it as being in large measure due to a therapeutic effect on the rate of progression of cancer that is already established, but still undiagnosed. But there is no good reason to believe that a therapeutic effect of high Se intake on the rate of progression of cancer should be much less in late rather than in very early stages of the disease.

#### Is it good prophylactic health policy to use dietary supplements to compensate for the consequences of low dietary intake of Se and unnatural fatty acid composition of poultry and swine meat?

If one wishes to reduce the burden of disease at a population level, increasing the Se intake through the ordinary diet may be a better strategy, rather than improving Se status on a more individual basis by use of Se pills. One reason for this is that it is far more expensive for the customer to buy Se or other nutrients in form of pills rather than getting them through the ordinary diet. It also depends on the level of education and knowledge about health-related issues whether or not people will buy such dietary supplements that are thought by nutrition experts to be good for their health. Both factors will favour those individuals and families who have the best education and most money - which means that the distribution in form of dietary supplements (rather than through ordinary foods) of such nutrients (like Se) that might be deficient in the ordinary diet can not be expected to be much helpful for those socioeconomic groups who now have the biggest health problems, *e.g. *because of smoking or too much alcohol in combination with poor diet. However, if the same nutrients could come through commonly eaten foods such as poultry meat, pork and eggs, it means that one can reach also many of those people who either can not afford to buy much dietary supplement preparates or are not well enough informed about health questions in general that they understand why it might be useful to take them.

The same can be said for the long-chain *omega*-3 fatty acids EPA and DHA, where intake through ordinary foods will also be a better strategy for reaching the entire target population, at the same time as intake through ordinary foods will be associated with less risk of EPA and DHA peroxidation during storage, compared with fish oil capsules (since rancidification of foods is very easily detected by consumers and an important reason for not eating the food, while the same may not be the case for fish oil inside a capsule).

Also when EPA and DHA come from animal foods rather than as purified dietary supplements, they will be ingested together with antioxidant nutrients that are important for prevention of peroxidation *in vivo *(following ingestion), such as Se, GSH (plus GSH precursor amino acids), carnosine and taurine. These nutrients can because of their antioxidant properties protect against tissue damage caused by ischemia and reperfusion [154,252,255,272-277] (in which respect they may very likely interact synergistically with each other, even if such interactions may still not have been much systematically studied either through animal experiments or clinical trials), and also against oxidative and nitrative stress-mediated tissue damage caused by severe infectious diseases, like hypervirulent avian influenza [90,122,154]. Some of them (if not all?) have also important antimutagenic [278], anticarcinogenic [279,280], and anti-inflammatory [154,281-283] properties in their own right. They may thus very likely synergize with many of the protective effects of long-chain *omega*-3 fatty acids, both in patients suffering from cardiovascular diseases, in patients suffering from skeletomuscular diseases leading to ischemic muscle pain and in patients suffering from chronic non-infectious inflammatory diseases, such as rheumatoid arthritis or Bekhterev's disease.

#### Optimizing the composition of poultry and pork meat is practically feasible and can be expected to lead to significant health improvement at a population level

By adding Se-enriched yeast to the chicken or pig feed concentrate, a meat that contains the same amount of Se as in fish, or even higher, can be produced [18,266,284]. The fatty acid metabolism in Se-deficient animals has earlier been shown to result in reduction of the concentrations of long-chain fatty acids such as DHA and other long chain C20 and C22 polyunsaturated fatty acids in rats [285]. The reason is not known for certain, but there is strong reason to expect that Se deficiency will lead to more rapid degradation of long-chain polyunsaturated fatty acids by lipid peroxidation. In a previous study in broilers given a relatively high Se intake through the feed (0.50 mg/kg diet or 0.84 mg/kg diet), the highest Se intake resulted also in increased concentration of EPA, DPA and DHA in the broiler meat [18].

It has been shown that by adding rapeseed oil and linseed oil to chicken diets, one can approximately double the concentration of very long chain fatty acids in meat when compared to regular chicken thigh meat [14,18,41]. Thus, broiler meat may give a significant contribution to the dietary intake of EPA, DPA and DHA and be close to rival lean fish such as cod in its content of the very long chain *omega*-3 fatty acids. Fatty fish such as salmon, herring or mackerel is still a much better source of these fatty acids. An experimental diet containing 40 g rapeseed oil and 10 g linseed oil per kg diet gave a broiler meat having a favorably low ratio between total *omega*-6 and *omega*-3 fatty acids; being about 2:1 and the ratio between AA and EPA about 3:1 [41]. This ratio, as earlier explained, is important for several reasons, *i.a. *since *omega*-6 and *omega*-3 fatty acids compete with each other for binding to enzymes and incorporation into membrane lipids [68], and *omega*-3 fatty acids can also suppress the expression of inflammatory genes [70,71], whereas *omega*-6 fatty acids have an opposite effect [70]. In addition, the ratio between *omega*-6 and *omega*-3 also influences several processes at the cellular level including cell growth, multiplication, apoptosis and cell survival [70], that might potentially be important especially for cancer patients.

There are, moreover, also recent observations suggesting that a high dietary intake of EPA and DHA can modulate the energy metabolism of adipocytes (by inducing mitochondrial biogenesis and *beta*-oxidation in these cells) in a way that might be useful for combating overweight and obesity [286]. Thus, it is possible that optimizing the *omega*-3/*omega*-6 fatty acid ratio of animal foods also might be useful as one of the components in a multifactorial strategy to combat the epidemic of obesity now being one of the world's major public health problems.

#### Can changes in sulphur amino acid/GSH status modulate pain sensitivity, mood and appetite regulation by changing the rates of prostaglandin, dopamine and serotonin biosynthesis in the central nervous system?

Other observations [287,288] suggest that the GSH concentration in the mammalian brain (including the brain of humans) may be very poorly homeostatically controlled. The affinity of the transport system for GSH is so low, at least in the rat [287], that the rate of GSH transport across the blood-brain barrier probably must be strongly dependent on the GSH concentration in blood plasma. At the same time, it is now very well documented that the rate-controlling enzymes in the synthesis of serotonin and dopamine in the brain, *viz. *tryptophan hydroxylase and tyrosine hydroxylase, are sensitive to oxidative and/or nitrative stress [289-291]. It is therefore possible that GSH depletion in the blood plasma, happening because the intake of sulphur amino acids (or other GSH precursor amino acids) is less than optimal (or because of too much alcohol or infavourable balance between hormones either enhancing or inhibiting glutathione synthesis in the liver), directly may lead to depression of serotonin and dopamine biosynthesis in the brain (because the two rate-controlling enzymes are both inhibited as a consequence of impairment of the antioxidative and antinitrative defense systems when brain GSH is depleted). This might conceivably contribute to enhancement of psychiatric problems like morbid depression and anxiety, enhanced irritability and poorly controlled aggressive behaviour. It might, moreover, be speculated that it also could play an important role in the etiology of disturbances of eating behaviour, not only when the main problem is hyperphagia leading to overweight, metabolic syndrome and obesity, but perhaps also when it is the opposite, *viz. *anorexia nervosa.

Given the importance of prostaglandin synthesis not only peripherally, but also inside the central nervous system for control of pain sensitivity [82], and given also the importance of GPx and its reducing substrate GSH for controlling the rate of the COX reactions, as earlier explained, there may be good reason to ask what is the effect of changes in the brain GSH concentration on pain sensibility, as well as on the control of fever. Is it possible that depletion of GSH in blood plasma could be one of the main causes of enhanced pain sensibility in patients that suffer from protein catabolic conditions with strong enhancement of the rate of cysteine degradation to sulfuric acid - *e.g. *in patients suffering from cancer cachexia? And it is possible that improvement of GSH status could have an antipyretic effect, *e.g. *in children with high fever, comparable perhaps to the effect of drugs that inhibit prostaglandin synthesis in the central nervous system?

These are important arguments why it might be better from a public health point of view to optimize the composition of commonly eaten animal foods rather than recommending to the whole population or to special groups of patients (*e.g. *with cancer) to restrict their consumption of meat. There are, however, still very important global ecological reasons to restrict the total consumption of animal foods, especially in the more affluent countries. But it is important, if animal foods shall be substituted with plant protein foods more than now, that this can happen in a way not leading to deterioration of the quality of the total diet, *e.g. *because of reduction of the intake of sulphur amino acids (as may happen if animal protein is replaced by soybean products or other legume protein with low concentrations of S amino acids).

#### Different methods can be used to optimize the fatty acid composition of animal foods

We believe that the broiler meat in the study referred to above [41] from a nutritional point of view (for improving the health of the consumers and reducing the burden for society from expensive, but entirely preventable diseases) is a much better alternative to regular broiler meat. We believe also that it is a better alternative, especially for patients suffering from depression, from alcohol abuse, from eating disorders or from infectious diseases, chronic non-infectious inflammatory diseases (such as rheumatoid arthritis, psoriatic arthritis and Bekhterev's disease) and other protein catabolic conditions (*e.g. *cancer cachexia), to supplying the same nutrients in form of dietary supplements (such as Se pills and fish oil capsules). One important reason for this is the synergistic interaction not only between long-chain *omega*-3 fatty acids and Se, but also between these substances and GSH not only in relation to prostaglandin and thromboxane biosynthesis, but also in relation to other physiological mechanisms relevant for cardioprotection (such as anti-ischemic protection and prevention of abnormal electrophysiology in cardiomyocytes), for reducing the rate of cancer progression, and for reduction of pain and harmful chronic inflammation. But it is possible that an optimal feed mixture for producing a specifically tailored "functional food" meat for patients suffering from cancer or severe chronic inflammatory diseases (such as rheumatoid arthritis or Bechterev's disease) should contain even more linseed oil and less rapeseed oil, so as to bring the ratio between *omega*-6 and *omega*-3 fatty acids in the meat even lower.

It is in principle easy to imagine other methods than those we have used for optimizing the *omega*-6/*omega*-3 fatty acid ratio of poultry meat, pork meat and eggs. An obvious alternative to adding *omega*-3-rich plant oils or seeds to the feed mixtures is to give the animals much more green leaves than they commonly receive in modern industrial farming systems. While this may not be difficult for small-scale farmers practising various forms of old-fashioned or more modern forms of organic agriculture, it might still be premature to have any firm opinion whether or not it might also be practically and economically feasible for modern industrial-scale farmers, or what might be the best practical methods for the latter if they wish to do it. Could we use grass-meal stored under inert gas as part of the feed mixtures given to poultry or pigs? Or would it be profitable for farmers to start growing plant species now regarded only as weeds for use as chicken feed? We are sorry that we can not for the moment give any good answer to any of these questions.

##### We need new and better regulatory standards for the composition of all types of meat

We have ourselves been working with broilers rather than with pigs mainly for the simple reason that the broiler is a much cheaper experimental animal compared with the pig. But there is no reason to believe that the same principles can not also be applied to the production of swine meat, or to the production of meat from other species of birds, such as turkey, duck, goose or quail. We believe it is also important to think about the overall fatty acid composition of the feed during production of ruminant meat, especially from beef cattle, and that regulatory standards should be imposed by law, requiring that the *omega*-6/*omega*-3 concentration ratio of meats, offal and eggs from all species (including both ruminants, monogastric mammals and birds) should not be much higher than might be considered natural for the species concerned. It is also possible that new regulatory standards should be imposed regarding conditions during storage and transport both for animal feed products and for meat, so as to ensure better protection against peroxidation than now for such more *omega*-3 fatty acid-rich products that we desire, at the same time as the human consumers should be much better protected than now against all such synthetic antioxidants that can have mutagenic and/or carcinogenic effects in mammals.

### Health economy

Meat from chicken and swine can be improved by feeding strategies to contain less AA and more EPA, DHA and Se. This review shows the potential health benefits of such meat. But what would be the estimated cost compared with the economic gain for society as a whole, in the hypothetical case that the same principles should be imposed by law for the entire agricultural sector in countries such as Norway, the United States, India, Russia or China?

We will not try to answer this question here, as we feel it goes far beyond our own respective fields of scientific competence, at the same time as the question is such a large one that it would probably not be possible even for a very well qualified health economist and statistician to answer it in the space of a short article. We can, however, give a concrete example for illustrating one small part of the problem: The consumption of analgesic (painkiller) medications is increasing worldwide, and in Norway the average person spends annually about 100 kroner to buy analgesic drugs (Legemiddelforbruket.no). The extra cost of producing meat that is high in Se and also has a low AA concentration and a favorably low ratio between *omega*-6 and *omega*-3 fatty acids would also be in this same price-range per person per year. But huge benefits in the health and social budgets from reduction in sick leaves, early retirement (*e.g. *because of painful skeletomuscular diseases) and consumption of hospital and primary health care services may be predicted. There would also be important life-quality benefits not easily measurable for economists as it might give less pain and suffering, and an improved all-over health (considering both somatic and psychiatric morbidity) that many people will consider to represent a priceless gift. Hopefully, it might also help to reduce the incidence over the next few decades of chronic diseases, such as asthma, that are associated with the use of the analgesic drug acetaminophen, at the same time as it is also possible that it might help to protect future generations against acetaminophen-induced or other drug-induced mutations.

So if we take only into consideration the problems of pain and of pain therapy, it is not unreasonable that there might be a net economic gain for society as a whole, if all poultry and swine farmers were required by law to make only products with an *omega*-6/*omega*-3 fatty acid ratio not higher than 2/1 and a Se concentration similar to what we find in marine fish (which would be expected to synergize with a reduction of the AA/(EPA + DPA + DHA) ratio of the meat for checking the problem of eicosanoid overproduction in many disease situations). If other diseases (such as cancer and sudden cardiac death) are also taken into consideration, it must be expected that the calculated health economic gain (as based on the best available statistical data) would be much larger.

### We need a better integration of human nutrition science and human pharmacology, and of agricultural science with medical science

During those discussions taking place during World War 2 that preceded the foundation of the FAO, one of the most important hopes and aspirations was that the new organization should help to solve important health problems related to scarcity of food or inadequate nutritional quality of the diet for large groups of people [292,293], not only in the poor countries (or colonies) in Africa, Asia and Latin America, but even in some of those countries that were then among the most affluent ones in the world. This had its background not only in the war-time experience of how serious consequences starvation and severe malnutrition can have, and in the need for economic reconstruction in Europe following the end of World War 2, but also in what had happened during the economic crisis of the late 1920s and the 1930s, when farmers in North America were not able to sell much of the cereal grains that they had produced (which therefore instead were burnt) at the same time as large groups of people were undernourished, if not starving, and infectious diseases including tuberculosis were taking a heavy toll among undernourished or poorly nourished people.

The Australian government official and 'amateur' economist Frank L. McDougall had advocated a 'nutrition approach' to world agriculture and its extension into 'economic appeasement' already during the 1930s, and was frequently using the slogan 'to marry health and agriculture' [293]. Some people had hoped that the ideas expressed in this slogan could be realized even at the organizational level when the United Nations and a family of related organizations (including WHO, FAO, WFP, UNICEF and UNESCO) were founded following the end of World War 2, *i.e. *that the same organization might work both with health problems and agriculture (personal communication from Arne Løchen, who during the 1970s was director in the Norwegian National Nutrition Council/Norwegian FAO Committee). This did not happen, but the first Director General for the FAO, Lord Boyd Orr, was by education a medical doctor.

The problems discussed in this article illustrate how serious consequences it can have when exactly the opposite thing happens and health and agriculture (including the feed industry and food industry sectors) become totally divorced from each other. The companies selling feeds to the poultry and pig farmers today have presumably no idea what their products can do to the health of human consumers of poultry and pork meat, while a vast majority of medical practitioners prescribing acetaminophen, NSAIDs or COXIBs to their patients are probably equally as much ignorant about the way the fatty acid composition of common animal foods has changed historically, compared to the natural composition of the same products (from the same animal species when living in their natural habitats) and how this may affect the pain or other important disease symptoms suffered by their patients.

It may be possible, though, that the most serious problem here is not the lack of good enough communication between the community of medical practitioners and scientists on one side and practitioners and scientists in the agricultural sector on the other, but rather an absence of good enough integration within medicine itself, especially as a consequence of poor communication between human nutrition scientists and human pharmacologists.

In an essay published in 1948 [294], the British neurologist Walshe draws an analogy between the development of medical science and the evolution of the central nervous system. He quotes the great neurobiologist Sherrington (1857-1952), who says about the evolution of the central nervous system in vertebrates that "integration keeps pace with differentiation". When as a consequence of evolutionary change sensory organs become better developed than before, and also those parts of the central nervous system that process information from the sensory organ concerned, there will simultaneously be an expansion of the volume of such parts of the brain that help to integrate information from that particular sensory organ with information coming from other sensory organs. This, says Walshe, is how it also ought to be in medical science. But it is not how the situation actually is.

Walshe's book was published in 1948, when the volume of medical science (as measured both by the number of scientists and the total number of new publications per week) was vastly smaller than today, and likewise the volume of the cumulative results of all medical research until then as measured by the total amount of valid observations available in the bookshelves of university libraries (or today also in literature databases). The total amount of specialized diversity among medical scientists is also much larger today than it was in 1948, if we use a definition for diversity analogous to the definition that palaeontologists and evolutionary biologists use to quantify biological diversity, *i.e. *as measured by the total number of subfields with small groups of scientists that communicate well with each other, but to a much more limited extent communicate with the rest of the worldwide community of medical scientists.

It is of little use, when finding a serious problem, to try to look after individuals or groups of people to be blamed, unless a correct diagnosis of the problem also can make it easier to find a solution. This is no less the case when one is dealing with typical 'system errors' or 'system failure', as may to a large extent be the case here, than when individuals (*e.g. *the director of a company or some prominent politician) are to blame. A strong plea can, however, be made for much better integration between agricultural science and human pharmacology (but also within the medical science community itself between human pharmacology and human nutrition science) than we commonly see today. It is necessary for the pharmacologists, taken as a group, to learn much more than now about nutrition science, but it is also important for the human nutrition scientists, collectively speaking, to learn much more than now about human pharmacology and the mechanisms of action of some of the most commonly used drugs.

It is not easy, however, to see how the present situation may be allowed to continue, if it shall be possible for the world to mobilize those economic and manpower resources that we need for simultaneously handling the serious challenges in the health sector (in affluent and poor countries alike) and averting unprecedented environmental disaster caused by ourselves.

## Conclusion

We believe that making meat high in Se and EPA and DHA and low in AA is a good strategy for increasing the dietary intake of Se and very long chain *omega*-3 fatty acids and for correcting the dietary balance between *omega*-6 and *omega*-3 fatty acids rather than relying on dietary supplements in form of Se pills and fish oil capsules, or on fish resources that are already overexploited and can not cover EPA and DHA requirements (for ensuring optimal health) for more than a fraction of the world's total population. A reduction of the dietary level of AA can only be obtained by reduction of the AA concentration in animal products, or by drastically reducing the dietary intake of such products that now contain too much AA.

The combination of inadequate intakes of EPA and DHA with overconsumption of AA is now one of the major causes of high rates of cardiovascular death in many of the 'old' industrial countries, and there is reason to fear that some of the countries in Asia with rapidly growing economies may soon follow because of the modernization processes affecting much of the animal food production in those countries as well. At the same time, it is likely that a high dietary AA/(EPA + DPA + DHA) ratio also may lead to more rapid development of most cancers, especially in such cases where the tumour cells are expressing COX-2, and to aggravation of several chronic pain conditions and chronic inflammatory diseases. AA comes nearly exclusively from animal foods, and the best strategy for reducing the average AA intake at a population level is to make it mandatory for the farmers (and for the feed industry) that animal products shall have an *omega*-6/*omega*-3 fatty acid ratio that must not be higher than what might be considered natural for the species concerned (when the animals live in their natural habitats). Since endogenous synthesis of AA from LA is also substantial (and similar to the average intake of AA from the diet), we believe it may be almost equally important also to reduce the average LA intake from edible fats and oils, compared to the situation in several countries today.

Perhaps even more serious than the premature death of aging individuals from heart disease or cancer are those health problems that may develop as a consequence of overutilization of mutagenic drugs among young individuals who suffer from such non-lethal conditions that are nevertheless important as causes of pain. It is possible that acetaminophen (paracetamol) as a consequence of the pattern of localization of acetaminophen-metabolizing enzymes inside the cells, at least in the brain, may act mainly as a mitochondrial mutagen (with much less effect on the DNA molecules in the cell nucleus). If so, it is possible that acetaminophen may be relatively unimportant as a contributory cause of cancer, while it might be very important as a cause of premature aging of mitochondrial DNA both in the brain and certain other organs.

It is possible that such mechanisms might help to explain the epidemiological association that has been observed between acetaminophen consumption and asthma. The role of paracetamol as a causative factor in other diseases has not been equally well studied by epidemiologists as in the case of childhood asthma. But what about degenerative diseases affecting the brain, such as Alzheimer's disease - is it possible that acetaminophen consumption could have a similar effect there as well? What about mood and behavioural disorders, where enhanced mitochondrial ROS production in the brain also might be harmful? And what about pancreatic *beta*-cells also expressing the acetaminophen-metabolizing enzyme CYP2E1 (which at least some of the pancreatic *beta*-cell lines grown *in vitro *do) [295]? Animal experiments have suggested that accumulation of mutations in *beta*-cell mitochondrial DNA could be an important part of the pathogenetic mechanism in type-2 diabetes [67]. Is it possible that acetaminophen also could play a role as one among several contributory causes of type 2 diabetes by enhancing the rate of mutagenesis in *beta*-cell mitochondrial DNA? For these questions, we have still no final answers. But there must be ample reason, especially when combining all the good epidemiological studies linking acetaminophen and asthma with what is already known about the details of the metabolism of this drug (not only by cytochrome P_450_, but also by cyclooxygenases), to be extremely worried.

Liberalizing the distribution and consumption of a cheap, but mutagenic pain-killing drug is obviously not any good alternative to offering good prophylaxis or therapy for the disease causing the pain, or for normalizing the function of hypersensitive pain fibres. Still, this is exactly what the world is doing today. There might be more than one reason for this. But the most important one is probably also here the fragmentation and lack of integration of medical research knowledge, both when considering the communication between medical and agricultural scientists, between different subgroups of medical scientists and between all the scientists on one side and the medical practitioners plus the health and agricultural (plus pollution control) authorities in perhaps most countries in the world on the other.

A substantial share of the world's population lives in such poverty that their consumption of animal foods is very low. For these people, it must be expected that endogenous synthesis of long-chain PUFAs from LA and ALA must be much more important than the intake of long-chain PUFAs as such from the diet. If the LA/ALA ratio in the diet is then too high, it must be expected to have all the same adverse consequences as when the dietary AA/(EPA + DPA + DHA) ratio for more affluent people living on an ordinary mixed diet is too high. We believe therefore that regulatory standards should be imposed by law, regarding the ALA/LA ratio of edible fats and oils, especially for ensuring adequate supplies of DHA for incorporation into the lipids of the brain, retina and testicles in foetuses and children not only in affluent families, but also in the poor ones all over the world.

Of all health problems that have been discussed in this article, none is more serious than the problem of enhanced mutagenesis in germline cells, and the possibility that wrong fatty acid composition of the membrane lipids of male germ cells could be an important cause of enhanced mitochondrial ROS production enhancing in turn not only the rate of mitochondrial DNA mutagenesis, but also the mutation rate in the nuclear DNA of these cells. In this case, we are dealing with a potential menace even to the survival of our species. Our descendants can not afford complacency with the present state of affairs. Our attitude should not be, as was said in pre-revolutionary France: *Après nous, le déluge! *It is time for the medical world to wake up, if it shall be possible to save the planet, including ourselves.

## Competing interests

The authors declare that they have no competing interests.

OAC and AH were writing this manuscript. Both authors read and approved the final manuscript.
